# Supplementary material for: Selection of Reference Genes in Evodia rutaecarpa var. officinalis and Expression Patterns of Genes Involved in Its Limonin Biosynthesis
Source: Plants (Basel). 2023 Sep 7;12(18):3197. doi: 10.3390/plants12183197 (PMC10534417; doi:10.3390/plants12183197)
Supplement: Supplementary file 1 [file plants-12-03197-s001.zip › plants-2577385-supplementary.pdf]

Supplementary Materials

Text S1: Sequences of candidate refernce genes.

Actin-1

ATGACTCAGATTATGTTTGAAACTTTCAATGTGACAGCAATGTATGTTGCCATCCAGG
CCGTTCTATCTCTATATGCCAGTGGTCGTACAACTGGTATTGTGCTGGATTCTGGTGAT
GGTGTGTCTCATACTGTGCCAATCTATGATGGATATGCCCTTCCCCATGCAATTCTTCG
TTTGGACCTGGCTGGTCGTGATCTCACTGATGCATTGATGAACATTCTTACTGAGAGA
GGGTACATGTTTACCACCACAGCCGAGCGGGAAATTGTCCGTGACATGAAGGAGAA
GCTTGCTTATGTTGCTCTAGACTATGAGCAAGAACTTGAGACTGCCAAGAGCAGCTCT
TCTGTTGAGAAGAACTACGAGCTGCCTGATGGCCAGGTGATAACCATTGGAGCTGAG
AGGTTCCGTTGCCCAGAAGTCCTCTTCCAGCCATTTCTCATCGGAATGGAAGCTGCCG
GTATCCATGAGACTACCTACAACTCCATCATGAAGTGTGACGTGGATATCAGAAAGG
ATCTGTATGGTAACATTGTGCTCAGTGGTGGTTCGACTATGTTCCCTGGTATTAGCCGA
CCGTATGAGCAAGGAAATCACTGCACTTGCTCCAAGCAGCATGAAGATTAA

Histone H3

ATGGCCCGTACTAAGCAAACTGCTCGTAAATCAACTGGAGGAAAGGCACCTAGGAA
GCAACTCGCTACCAAGGCCGCCTGTAAGTCTGCTCCCACAACCGGTGGAGTCAAGAA
GCCACACCGTTACCGCCCTGGAACTGTCGCCCTTCGTGAAATTCGAAAGTACCAGAA
GAGTACCGAGCTACTGATCAGGAAACTCCCATTCCAGAGACTTGTTCGTGAAATAGC
CAAGGACTTCAAGACTGATCTGCGTTTCCAGAGCCACGCTGTCCTGGCCCTACAGGA
GGCAGCCGAGGCTTACCTCGTCGGTCTCTTTGAGGACACTCCCCTCTGTGCTATTCAC
GCCAAGCGTGTGACCATTATGCCCAAGGATATCGAGCTTGCCCGTAGGATCCGCGGT
GAGCGTGCTTAA

UBQ

ATGCAGATCTACGTCAAAACCCTAACCGGCAAAACAATCACTCTCGAAGTTGAGAGT
TCCGACACTATTGATAACGTTAAGGCCAAGATCCAAGACAAGGAGGGACACCCTCCT
GATCAGCAGCGCCTCATCTTCGCCGCCAAGCAATTGGAAGACGGACGGACCTTGGCT
GACTATAATATCCCGAAGGAATCAACCCTTCATCTTGTCCTCAGGCTCCGTGGTGGCA
TGCAGATCTTCGTCAAAACCCTTACCGGCAAGACGATCACGCTCGAGGTTGAGAGTT
CAGACACGATCGATAACGTCAAGGCCAAGAATCAAGACAATGAGGGGATCCCACCA
GACCAGCAGACGCTGATCTTTGCCGGAAAGCAACTCGAAGACGGACGCACCTTGGC
CGACTACAATATCCAGAAGGAATCAACCCTTCATCTTGTCCTCAGGCTCCGTGGTGGT
ATGCAGATCTTCGTCAAAACCCTAACTGGGAAGACGATCACTCTCGAGGTGGAGAGT
TCAGATACTATCGATAATGTCAAGGCCAAGATCCAGGACAAAGACGGCATCCCACC
GGATCAGCAAAGATTGATCTTCGCCGGCAAGCAACTCGAAGATGGCCGTACTTTGGC
TGATTATTACATTCAGAAAGAGTCAACGCTCCATCTGGTGCTTCGTCTACGTGGTAGG
CATGCAGATTTTTGTTAA

GAPDH

ATGTTCGTTGTTGGTGTCAATGAGCACGAGTACAAGCCAGAACTTAACCTTGTGTCCA
ATGCTAGCTGCACCACCAACTGCCTTGCTCCGCTCGCCAAGGTCATTCATGACTGGTT
TGGCATTGTTGAGGGTTTGATGACCACTGTTCACTCTATCACTGCCACCCAGAAAACT
GTTGATGGGCCATCATCAAAGGACTGGAGAGGTGGCAGGGCTGCTTCATTCAACATC
ATTCCTAGCAGTACTGGAGCTGCTAAGGCTGTTGGAAAGGTCTTACCTTCTTTGAATG

GAAAACTGATTGGTATGGCTTTCCGTGTACCCACGGTTGATGTCTATGTGGTTGACCT
CACAGTGAGGATGGAGAAGGAAGCTTCTTATGATGAAATTAAAGCTGCCATCAAGG
AGGAGTCCGAGGGAAAACTAAAGGGTATCTTGGGATACACTGAGGATGATGTGGTTT
CTACGGACTTTATTGGCGACAACAAGTCAAGTTTCTTTGATGCCAAGGCTGGTATTGC
TTTGAGCAAGAATTTTGTGAAGCTTGTCGCGTGGTATGACAATGAGTGGGGCTACAGT
TCCCGTGTGGTTGATCTGATTGTCCACATGTCGAAGACCCAGTAA

UBQ 10

ATGCAGATCTTTGTAAAGACCCTCACTGGAAAAACTATCACCCTTGAGGTGGAAAGC
TCTGACACCATTGACAATGTCAAAGCCAAAATTCAGGACAAGGAAGGGATCCCACC
AGACCAGCAAAGGCTCATCTTTGCCGGAAAACAACTTGAGGATGGACGCACACTTGC
CGACTATAACATCCAGAAGGAATCTACCCTTCATTTGGTTCTTCGTCTCAGGGGTGGC
ATGCAGATTTTCGTGAAAACCCTGACTGGTAAAACTATAACTTTGGAAGTTGAAAGCT
CTGACACGATTGACAATGTCAAGGCTAAGATCCAGGACAAGGAGGGCATCCCACCA
GACCAGCAGAGGTTGATCTTTGCCGGCAAACAACTTGAGGATGGCCGTACTCTTGCT
GACTACAACATTCAAAAGGAGTCAACCCTTCACCTGGTGCTCCGTCTCCGTGGTGGTA
TGCAGATCTTTGTGAAAACCCTCACTGGAAAAACAATCACTCTTGAGGTTGAAAGCT
CTGACACCATTGACAATGTTAAAGCAAAAATTCAAGATAAGGAGGGTATACCACCA
GATCAGCAGAGGCTTATTTTTGCTGGAAAGCAGCTTGAAGACGGAAGGACCCTTGCA
GACTATAACATCCAGAAAGAATCCACGCTACACTTGGTTCTTCGCCTCCGTGGTGGTA
TGCAGATTTTTGTGAAGACCCTCACTGGGAAAACCATCACCTTGGAGGTTGAGAGTTC
AGACACCATTGACAATGTTAAGGCTAAGATCCAGGATAAGGAGGGGATTCCACCGG
ACCAGCAGAGGTTGATCTTTGCTGGAAAGCAACTGGAGGATGGAAGTACTCTTGCTG
ATTACAACATCCAGAAGGTGTCCACTCTGCATCTTGTTCTTCGTCTCCGTGGAGGTTTC
TGA

CHS 1

ATGCTGACCGTCGATGAAGTTCGCAAGTCTCAACGTGCCGTCGGTCCGGCCACGATC
ATGGCCATCGGAACGGCAACTCCTCCGAACTGTGTTGACCAAAGCACATATCCTGAC
TATTATTTTCGTATCACAAAGAGAGAACACATGACTGAACTTAAAGAGAAATTTAAG
CGCATGTGTGACAAATCCATGATCAAGAAGAGATACATGTACTTGACTGAAAATATA
TTGAAAGAAAACCCTAATGTGTGTGAATACATGGAACCGTCAATAGATGCTAGGCAA
GATATGGTTGTAGTTGAGGTACCAAAGCTAGGCAAAGAAGCAGCCACCAAGGCCATT
AAAGAATGGGGGCAGCCCAAATCCAAAATCACCCACTTGGTCTTTTGCACTACTAGT
GGTGTTGATATGCCTGGGGCCGATTACCAACTCACCAAACTCTTGGGCCTTCGCCCTT
CCGTTAAACGTCTCATGATGTACCAACAAGGCTGTTTCGCCGGCGGCACGGTGCTCCG
TCTTGCCAAAGACCTCGCCGAGAACAACAAAGGCGCCCGTGTCCTCGTCGTGTGCTC
TGAGATAACTGCAGTTACTTTCCGGGGTCCCAGTGACACCCATCTTGATAGCCTTGTA
GGCCAAGCTTTGTTTGGTGACGGTGCTGCTGCTATTATTATTGGCTCTGATCCTGTTCA
ACGGCTCGAAAAGCCTATCTTTGAACTGGTCTCTGCGGCCCAAACAATATTACCCGAT
AGTGAAGGGGCTATTGATGGACACCTCCGTGAAATTGGGCTTACGTTTCACCTACTCA
AGGATGTTCCTGGGCTAATTTCGAAGAACATCCAAAAAAGTCTCACTGAGGCATTCA
ACCCATTGGGTATCTCGGACTGGAACTCGATTTTCTGGATAGCGCACCCCGGTGGGCG
TGCTATTTTGACCAGGTTGAGGAGAAGTTGGGCCTGA

β-TUB6

ATGAGAGAAATCTTGCACATTCAAGGAGGCCAATGCGGCAACAAAATCGGTTCCAA

ATTCTGGGAGGTCATTTGCGACGAGCATGGCGTGGACCCCACCGGGAGGTACAGAGG
AGACGCTGACTCGGATCTTCAATTGGAGCGGATCAATGTCTACTACAATGAAGCTTCC
GGTGGAAGGTACGTGCCTAGGGCTATCCTCATGGATCTTGAACCCGGTACTATGGAT
AGTATCAGATCTGGACCCTACGGCCAGATCTTTCGACCTGATAACTTCGTCTTTGGGC
AATCCGGTGCCGGCAATAATTGGGCCAAAGGTCATTACACCGAAGGCGCTGAGTTGA
TCGACGCTGTGCTCGACGTCGTTCGTAAGGAGGCTGAGAATTGTGATTGCTTGCAAGG
TTTTCAGGTTTGCCACTCACTTGGAGGAGGTACAGGTTCTGGCATGGGAACTCTCTTG
ATATCAAAGATTAGAGAGGAATATCCAGACAGGATGATGATGACTTTCTCTGTCTTCC
CTTCTCCTAAGGTCTCTGACACAGTCGTGGAGCCATACAATGCAACCCTTTCTGTGCA
TCAGTTGGTTGAGAATGCTGATGAATGCATGGTTCTTGACAATGAAGCACTTTATGAT
ATTTGCTTCCGGACTCTAAAGCTTAGCACTCCAAGCTTCGGAGACTTGAATCACTTGA
TTTCTGCAACTATGAGTGGTGTCACATGCTGCCTGAGGTTCCCAGGTCAACTCAACTC
TGACCTCAGGAAGCTGGCTGTTAATCTGATTCCGTTCCCACGTCTTCATTTCTTCATGG
TGGGATTTGCACCGCTCACCTCATATGGATCCCAGCAATACATCTCCCTCACTGTGCC
CGAGCTGACTCAGCAAATGTGGGATGCCAAGAATATGATGTGCGCAGCTGACCCCCG
CCATGGCCACTACCTCACAGCCTCAGCTATGTTCAGAGGAAAGATGAGCACCAAAGA
GGTGGATGAGCAGATGATTAATGTGCAGAACAAGAATTCATCATACTTTGTCGAGTG
GATCCCCAACAATGTGAAGTCCAGTGTGTGTGATATTCCACCTAGGGGTTTGAAGATG
GCATCTACTTTCATTGGTAACTCCACGTCTATCCAAGAGATGTTCAGGAGGGTGAGTG
AGCAGTTTACTGCCATGTTCCGGCGCAAGGCCTTTTTGCATTGGTATACTGGGCGAGG
GTATGGATGA

α-TUB

ATGCTGTTGGAGGTGGTACTGGTTCTGGACTCGGGTCACTTCTCTTGGAACGACTCTCT
GTTGACTATGGCAAGTAAATCAAAGCTTGGTTTCACTGTGTATCCTTCACCTCAGTTTT
CCACATCAGTTGTGGAGCCTTACTACAGTGTCCTTTCAACCCATTCCCTCCTTGAGCAC
ACCGATGTTGCTGTGCTTCTTGATAATGAAGCCATCTATGACATTTGTAAGCGTTCTCT
TGACATTGAGCACCCCACTTACACCAATCACAACCGATTAATCTCTCAGGTGATTTCA
TCCCTCACTGCTTCTTTGAGGTTTGATGGAGCTCTGAATGTGGATGTGACCGAGTTCCA
GACCAATCTGGTTCCCTACCCGAGGATTCATTTCATGCTTTCCTCTTATGCTCCAGTTA
TTTCTGCGGAGAAGGCATACCATGAGCAACTTTCAGTTGCTGAGATCACCAACACTG
CGTTTGAGCCATCATCCATGATGGCCAAATGTGATCCTCGTCATGGAAAATACATGGC
TTGTTGCTTGATGTTTCGTGGTAATGTTGTGCCTAAGGATGTGCATGCAGCTGTGGCCA
CCATCAAGACGAAGCAGACAGTCCAGTTTGTTGATTGGTGTCCTACTGGATTCAAGTG
CGGTATCAACTAA

Actin-100

ATGACCCAGATAATGTTTGAAACCTTCAATGTGCCTGCTATGTATGTTGCTATCCAGG
CCGTGCTTTCCCTTTATGCCAGTGGTCGTACAACAGGTATGGTCTTAGATTCTGGTGAT
GGTGTCAGCCATACAGTACCAATTTATGAAGGCTATGCGCTCCCTCATGCTATCCTTC
GTTTGGACCTTGCTGGCCGTGATCTAACGGACTCCTTAATGAAGATCCTCACTGAGAG
AGGGTATTCTTTCACCACTACTGCTGAGCGGGAAATTGTCCGTGACATGAAGGAAAA
ACTTGCGTATGTTGCCCTTGATTATGAGCAAGAGTTGGAGACTGCAAAGAGCAGCTCT
GCGGTTGAGAAAAGTTATGAGTTGCCTGATGGGCAGGTGATTACAATCGGAGCTGAG
AGGTTCCGTTGCCCAGAAGTACCTTTCCAGCCATCTTTGATTGGAATGGAAGCTGCTG
GAATTCATGAAACCACTTACAACTCCATCATGAAGTGTGATGTTGATATCAGGAAAG

ATCTGTTGGAAACATTGTGCTTAGTGGTGGTTCAATTATGTTCCCTGGTATTGCTGATC
GGATGA

β-TUB2

ATGGGAACCCTGTTGATATCAAAGATCAGAGAGGAATATCCTGACAGGATGATGATG
ACTTTCTCCGTCTTCCCTTCTCCAAAGGTCTCTGACACAGTCGTGGAGCCATACAATG
CAACCCTTTCTGTGCACCAGTTGGTTGAGAATGCTGATGAATGCATGGTTCTTGACAA
TGAAGCTCTTTATGATATTTGCTTCCGGACTCTAAAGCTTAGCAATCCAAGCTTCGGA
GACTTGAATCACTTGATTTCTACAACTATGAGTTGTGTCACTTGCTGCCTGAGGTTCCC
TGGTCAACTCAACTCTGACCTCAGGAAGCTGGCTGTTAATCTGATCCCATTCCCACGT
CTTCATTTCTTCATGGTGGGATTTGCACCTCTCACCTCACGTGGATCCCAGCAATACAT
CTCCCTCACTGTGCCCGAGCTGACTCAGCAAATGTGGGATGCCAAGAATATGATGTG
TGCAGCTGACCCCCGCCATGGCCGCTACCTCACAGCCACAGCTATGTTCAGAGGAAA
AATGAGCACCAAAGAGGTGGATGAGCAGATTATTAATGTACAGAACAAGAATTCAT
CATACTTTGTAGAGTGGATCCCCCACAATGTGAATTCCAGTGTATGTGATATACCACC
AAGGGGTTTGAAGATGGCATCTACTTTTATTGGTAACTCCACGTCTATCCACGAGATG
TTCAGGAGGGTGAGTGAGCAGTTTACTGCCATGTTCCGTCGCAAGGCCTTTTTGCATT
GGTATACTGGTGAGGGTATGGATGAAATGGAATTCACGGAGGCAGAGAGCAACATG
AATGACTTGGTGGCAGAGTACCAACAATAA

Text S2: Sequences of pathway genes.

DXS

ATGGCTCTCTCTGCATTCTCATTTCCAACTCACGTCGACAAAGCAACAATTTCAGTTCC
TCGAAAGTCTTCTCTGCTTACTTCTCATTTCTTGGGAGCTGATATTTTTTCCCATTTTTCT
CATAAGCTCATCAATCAGCAGATACAGCTCAAGAAAAGGCCAAATGGGGTTTGTGCA
TCACTTTCAGAAAGAGGAGAATATCACTCACAAAGACCACCAACACCTCTATTGGAC
ACCATAAACTATCCAATTCACATGAAAAATCTATCTATCAAGGAGCTTAAACAACTAG
CAGATGAGTTGCGGTCAGATGTCATAGTCAATGTTTCGAAAACTGGGGGTCACTTAGG
CTCAAGTCTTGGTGTGGTTGAGCTTACTGTGGCTCTTCATTATGTTTTCAATGCTCCTAA
AGACAGGATACTATGGGATGTTGGTCATCAGACTTACCCTCATAAAATCTTGACTGGG
AGAAGAAATAAGCTGCATACCATGAGGCAAACAGATGGCCTTTCGGGATTCACTAAG
CGATCTGAGAGTGAATATGATTGCTTTGGAACCGGCCACAGTTCCACTAGTATCTCTGC
TGGATTGGGTATGGCCGTGGGTCGGGATCTTAAAGGAAGAAGGAATAATGTGGTTGCT
GTTATTGGTGATGGTGCCATGACTGCAGGCCAAGCTTATGAAGCCATGAACAATGCTG
GGTACCTAGATTCTGACATGATTGTTATTCTTAATGACAACACACAAGTTTCTTTACCCA
CTGCTACTCTAGATGGGCCCATACCACCTGTCGGTGCTTTGAGCAGTGCTCTCAGCAG
GTTGCAGTCAAACAGGCCTCTCAGAGAACTTAGAGAGGTTGCAAAGGGAGTTACTAA
GCAAATAGGTGGGCCAATGCATGAACGGGCTGCAAAAGTTGACGAATATGCTCGTGG
GATGATCAGTGGTTCTGGGTCATCACTGTTTGAAGAACTTGGACTGTATTATATTGGTC
CTGTTGATGGTCACAATATAGATGACCTTGTTGCCATTCTTGAAGAGGTTAAGAGTACC
AAAACAACAGGTCCAGTTCTGATCCATGTTGTCACTGAGAAAGGCAGGGGATATCCAT
ATGCTGAAATGGCTACAGACAAGTACCACGGTGTGGCAAAGTTTGATCCAGCCACTG
GAAAGCAATTCAAATCCGGTGCCAGGACCCAGTCTTATACAACATACTTTGCAGAGGC
TTTGATTGCAGAAGCTGAGGTGGACAAGGATATTGTTGCTATCCATGCTGCAATGGGA
GGTGGAACAGGACTGAATCTCTTCCTTCGTCGTTTCCCTACTAGATGCTTTGATGTTGG
GATAGCAGAACAGCATGCTGTTACTTTTGCCGCTGGTCTGGCCTGTGAAGGCCTTAAA
CCTTTTTGTGCTATCTATTCGTCTTTCATGCAAAGGGCTTACGACCAGGTAGTACATGAC
GTGGATTTGCAGAAGCTGCCTGTAAGATTTGCAATGGACAGAGCTGGACTTGTTGGAG
CTGATGGTCCCACTCATTGTGGGTCTTTTGATGTCACTTTTATGGCGTGCCTTCCTAACA
TGGTGGTAATGGCTCCTTCTGATGAGGCAGAGCTTTTTCACATGGTTGCAACTGCTGCT
GCCATAGATGACCGGCCTAGCTGTTTCCGCTACCCAAGAGGAAATGGGATTGGTGTTG
AACTGCCACCAGAAAACAAAGGCACTCCCCTTGAGGTTGGGAAAGGCAGGATACTG
ATTGAAGGGGAGAGGGTTGCACTCTTGGGCTATGGGACAGCTGTTCAAAGCTGTTTAG
CTGCTGCTTCTTTATTGGAATCCCATGGGTTACGGCTGACTGTAGCAGATGCTCGATTCT
GTAAGCCACTGGATCATTCCCTCGTTCGTAGCCTGGCAAAATCACACGATGTTTTGATT
ACCGTGGAGGAAGGATCAATTGGGGGTTTTGGTTCTCATGTTGCTCAGTTCTTGGCACT
TGATGGTCTTCTTGATGGCACCATCAAGTGGAGACCACTCGTACTCCCTGATCGATATA
TCGATCATGGAGCACCTGCTGATCAGATAGCTCAAGCTGGTCTCACACCATCTCACATT
GCAGCAACAGTATTTAACATTCTTGGACAAACAAGAGAGGCTCTCGAGATAATGTCAT
TCAAAAACTAA

DXR

ATGGCTTTGAATTTGCTATCCCCAGCTGGAACCAAAGGTCTCTCTTTCTTGGATACCAC
GAGGTCCCATCACCGTCTCCCCAAGCTGTCAGGTGGGTTACCTTGGAAGAGGAAGGA
TAATGGAACGGTGACGTTTGGAAGAATAGTTCAATGTTCTGCTCAAGCACTTCCTCCA

GCGTGGCCAGGCCGAGCTGTTGCAGAATCCTTTCGTAAGACTTGGGATGGTCCAAAGC
CAATCTCAGTGCTCGGATCAACTGGTTCTATTGGTACTCAGACATTGGATATAATTGCG
GAACATGAAGATAAATTCAGAGTTGTGGCACTCGCAGCTGGTTCAAATGTTACTCTTC
TAGCTGATCAGGTGAAGAGATTCAAACCTAGACTAGTTGCTATCAGAAATGAGTCATT
ACTTGATGAACTCAAGGAGGCTTTGGCTAATGTTGAACACAAGCCTGAGATTGTGGCT
GGGGAGCAGGGAGTTCTTGAGGTTGCCCGACACCCAGATGCCGTCACCGTAGTGACA
GGGATAGTAGGTTGTGCAGGATTAAAGCCTACGGTTGCAGCAATAGAAGCAGGGAAA
GACATAGCGTTGGCCAATAAGGAGACCATGATTGCTGGTGGTCCTTTTGTCCTTCCTCT
AGCTCACAAGCATAATGTAAAAATTCTTCCAGCTGATTCAGAACATTCTGCCATATTTC
AGTGTATTCAAGGCCTGCCTGAGGGTGCACTTCGGCGTATTATTTTAACTGCATCTGGC
GGGCCCTTCAGGGAATGGCCGGCTGAGAAACTGAAAGAAGTTAAAGTAGCTGATGCA
TTGAAGCATCCTAACTTTACTCTGGGAAAAAAGACTACTGTGGAGTCTGCTACAGTTTT
CAACAAGGGTTTAGAAGTAATTGAAGGCCATTACTTATTTGGAGCTGAATATGATGATA
TTGAGATAGTAATTCATCCACAGTCAATCATACATTCAATGGTTGAAACACAGGATTCC
TCTGTTATTGGACAGTTGGGGTGGGCTGATATGCGCATACCACTCCTTTATACAATGTCT
TGGCCAGAAAGAATTTCCTCCTCTGAATTGACATGGCCTCGCCTTGATCTTTCCAAGCT
TGGTTCATTAACATTTATGGCTCCTGACACTGAAAAATTTCCATCCGTGAATCTTTGCTA
TGCTGCTGGACGAGCTGGAGGCACAATGACAGGAGTTCTTAGTGCAGCAAACGAGAA
AGCAGTAGAGATGTTCATCGATGAAAAGATTAGCTATCTGGATATTTTCAAGGTTGTGG
AGTTAACGTGTGATAAGCACCGGGCAGACTTGGTGACCTCGCCATCCCTGGAAGATAT
ATTACATTATGATGCGTGGGCACGAGATTACGCAGCTAGTTTGCAAGTTTCATCCAGTA
GAAGCCCTGTTCCGGCATGA

ISPD

ATGGGGCTTCTTCAACTGAACCTAGCTCTATCTTCTTCAACGTCCAACCACCAAACTTT
CTCTAGAACTGGTTATAAAAATAATTTCTTTGACTTGACAAGGAAGGCCAGGATTTCCA
TACCCAAGATTAGATGTTCTGCAAAAGTTGATAATGGTTCTGTTGTTGTAAAAGCTAAT
AGTGTATCAGTTATTCTTTTGGCTGGAGGAAAGGGCAAAAGAATGGGTGCGAGCATGC
CAAAGCAGTATCTACCCCTTCTGGGTCAACCGATTGCATTGTATAGTTTCTACACTTTCT
CGCGCATGATTCAAGTGAAGGAAATTGTGGTAGTTTGTGATCCATCTTACAGTGACATC
TTCGAATAA

ISPE,

ATGGCTATGGCGTCCTCTTATTTGCAGTTCAATCACCACCACCAACTCCATCTACCTTC
GATTAGCCCTAATGGGTTTTCTTCTTTCAAACCAAAAGTTCATTTTCGCAGGACCCAAC
TTGTCAAATGTACCAGAAAACAAGTCGAGATAGTGTATGATCGTGATGAAAGATTAAA
CAAGTTGGCAGATGAAGTAGACAGGGATGCTCCTCTTTCAAGGCTCACTCTGTTCTCA
CCTTGCAAGATAAATGCTTTTTTGAGAATTACAAACAAGAGAGAAGATGGGTATCATG
ATTTAGCATCTCTCTTTCATGTAATTAGTTTAGGTGATACAATTAAGTTTTCTTTGTCACC
GTTGAAGACTAAGGATCGTTTGTCAACCAATGTGGCAGGGATACCTCTTGATGACAAT
AATTTGATAATTAAGGCCCTTAACCTCTATAGAAAGAAGACAGGCAGTGACAATTTCT
TTTGGATTCATCTTGATAAAAAGGTGCCTAGTGGGGCAGGACTTGTTGGTGGAAGCAG
CAATGCAGCCACTGCACTTTGGGCAGCAAATCAATTCAATGGTTCTGTTGCCACAGAG
ACGGAACTCCAAGAATGGTCAAGTGAGTTTGGTTCAGATATTCCCTTCTTTTTCTCCCA
TGGAGCAGCTTATTGCACTTGTAGAGGTGAGGTTGTTCAAGATATTCCCCCACCCATAC
CTATGGATGTCCCAATGGTTCTTATAAAGCCCAAGGAGGCATGTCCCACGGGTGAAGT

GTACAAGCGTCTTCGACTTGATCAAACCAGTAAGGTTGATCCTTTAACATTGCTCGTGC
AGATTTCAAGGAACGGATTGACTCAAGATGTTTGTATCAATGATCTGGAAGCACCGGC
ATTTGAAGTTCTCCCATCTCTTAAAAGATTGAAACAGCGTATAATTGCAGCTGGCCGTG
GACAATATGATGCTGTTTTTATGTCTGGCAGCGGTAGCACTATTGTTGGCATCGGCTCC
CCAGACCCTCCACAGTTTATCTATGATGACAACGAGTATCATGATGCATTTTTGTCAGA
GGCTCGGTTTATCACTCGTGAAGTGAATGACTGGTATAGAGAACCCGCTTCGACAAAC
GATTGTAGCCAACCCCCTGCATTTTCCGGAACTGAGGGGTGA

ISPF

ATGTCCTCCTCCTTTGAGCCTCGTAGCTTATCCACCAAACATCTCCGGTCAACGTCGTT
GCCTCGTTTCTCAGTTTCAGTTTCAGCTGCAGCCGCAACTTCCTTCCAAGTCAAAGAGT
CATCGACTTCAATCCAAACGTCCAAATCAAAATCACTTCCTTTTCGTGTGGGCCATGG
GTTTGATCTTCACAGATTGGAACCTGGCTACCCTCTGATCATCGGTGGCTTCGATATTCC
CCACGATAGAGGCTGCGAGGCTCACTCTGATGGTGATGTTTTGTTACACTGTGTTGTGG
ATGCGATTTTGGGAGCACTGGGGCTTCCGGATATGGTCAGATATTTCCTGATAATGATC
CTAAGTGGAAGGGTGCACCATCCCCTGTTTTCATTAAAGAAGCTGTTAGACTCATGGA
TGAGTCAGGATATGAGATTGGCAACATGGCTGCCACGTTGA

ISPG

ATGAGGAATTCAAATTCCAGTTCGAATATCGCAGAGCTTCAGCCTGCATCAGAAGTTA
TTCCCCGACAAAAGTATTGTGAATCCATTTACAAAACAACCAGGCGGAAAACACGGA
CAGTGATGGTGGGAAATGTTGCAATGGGTGGTGAGCATTCTATTAGATTTCAGACAAC
GACTACAACGGATACTAAGGATGTTGCTGGAACAGTTGAACAGGTAATGAGAATAGC
TGACCTGGGAGCAGATCTTGTTCGGATTATAGTTCAAGGGAAGAGAGAGGCAGATGC
ATGTTTTGAGATAAAAAACTCACTTGTGAAGAAAAATTACAATATACCTCTGGCGGCA
GATATTCAATATTATCCCCCTTTTGCACTACAAGTTGTTGAATGCTTTGACAAAATCCGT
ATCAACCCAGGAAATTTTGCTGCTAGACGGGCTCAATTCGAGCAGCTAGTGTATACAG
AAGAGGACTATCAGAAAGTACTTGAGCATATTGAGGAGGTTTTTACTCCATTGGTTGA
AAAATGTAAGAAGTATGGAAGGGCAATGCGTATTGGGACAAACCGTGGGAGTCTTTC
AGATCGTATCATGAGTTGCTATGGGGATTCTCCTTGGGGAATGGATGGTTTGGGTGATA
TAATCAGGGTTTCGCTCACAGAAGCACCAGAGGAGGAGATATATCCTTGCAGAATTTT
GGCCAACCTGGGTATGAGAGCGCTGAGCTTCAGAAAGGAGTGGCACCATTTGAGGAA
AAGCACAGACAATATTTTGATTTTCATCGTCGTTCTGGTCAACTGCCAATTCAAAAGAA
GGGTGAGGAGGTGGATTACGGATTTGTTCTGCACCGTGATCGCTCAGTTCTCATGTCCG
TTTCTCTGGATCAGTTAA

ISPH

ATGGAGCTCATGAATCAAGAGTACACAAGTGATATTATAAAGATATTGAAAGAGAATG
GAAATGAGTATACTTGGGGGAATGTCACAGTGAAGCTGGCCGAATCATATGGTTTTTG
CTGGGGAGTTGAACGTGCCGTGCAGATTGCTTATGAAGCAAGAAAACAGTTTCCTGA
AGAAAGGATTTGGATTACCATTGAAATCATTCACAACCCGACTGTTAATAAGCGGTTG
GAGGAGATGGAAGATCAGAATATACCTGTCGACGAAGGAAAGAAGCAGTTAGATGTT
GTAAATAAGGGTAATGTTGTGGTTTTGCCTGCATTTGGGGCTGCAGTGGAGGAAATGG
TCACTCTAAATGACAAGAATGTACAGATTGTTGATACAACTTGCCGTTGGGTGTCTAAG
GTCTGGAATACTGTTGAGAAGCACAAGAAGGGAGACTACACTTCAATTATTCACGGTA
AATACTCTCATGAGGAAACTGTAGCAACTGCATCTTTTGCGGGAAAGTATATCATAGTA
AAGGATATGAAAGAGGCTTTGTACTTGTGTGATTACATTCTCGGGGGTGAACTTAATGG

ATCTAGCTTAACAAAAGAAGCGTTCCTTGAGAAATTTAAGAAAGCTGTTTCAAAAGG
GTTTGATCCAGAAATTGACCTGATAAAAACTGGCATTGCAAACCAAACTACAATGCTC
AAGGGTGAAACAGAAGAGATTGGAAAATTAGTTGAGAAGACCATGATGCGTCAAATA
TGGAGTGGAAAATGTCAACGAACACTTCATAAGCTTCAACACAATTTGTGA

ACAT

ATGAAAGCTACAATGCTTGCAGCACAGAGTATTCAGTTGGGGATCAATGATGTTGTTG
TGGCTGGAGGCATGGAAAGCATGTCTAACACTCCTAAATATCTGGCAGAAGCAAGGA
AAGGGTCTCGACTTGGACATGATTCTCTAGTTGATGGAATGCTTAAAGATGGGTTAAG
GGATGTTTATAATGATTGTGGCATGGGAACTTGTGCTGAACTATGTGCTGATCAACATT
CGATAACAAGGGAGGATCAGGATAACTATGCTGTTCAGAGCTTTGAGCGTGGCATTGC
AGCCCAAGATGCTGGTGCCTTTACATGGGAAATTGTTCATGTTGAAGTTTCTGGGGGA
AGGGGAAAACCGTCAACCATTGTTGATAAGGACGAAGGTTTAGGCAAGTTTGACCCG
GCAAAATTGAGGAAGCTTCGACCAAGTTTCAAGGATAATGGAGGAACTGTTACTGCT
GGCAATGCCTCTTGTATAAGTGATGGAGCTGCAGCATTGGTTCTAGTGAGTGGAGAGA
AAGCACTTAAGCTTGGTCTGCAAGTGATTGCGAAGATCACAGGATATGCTGATGCTGC
TCAGGCACCAGAGAACTTTACAACAGTTCCAGCCCTAGCAATACCAAAAGCTATTTCA
AATGCTGGATTGGAGGCTTCTCAAGTTGATTTTTACGAAATAAATGAGGCCTTTGCTGT
TGTGGCCCTTGCAAACCAGAAATTGCTCGGACTAAATCCTGATAAGGTTAATGTGCAT
GGTGGAGCTGTATCATTGGGACACCCTCTGGGTTGCAGTGGAGCTCGTATCTTGGTCA
CACTTTTAGGGGTGCTTAGGCAGAAAAATGGGAAATACGGTGTTGGTGGCGTTTGCAA
TGGAGGAGGTGGTGCATCGGCGCTTGTTTTAGAGCTTGTGTAA

HMGCS

ATGTCTCATGCCTATGATTTCTATAAGCCAAACCTTGCTAGCGAATACCCGGTTGTTGAT
GGGAAACTTTCTCAGACATGCTACCTCATGGCTCTTGATTCTTGTTACAAACAATTATG
TAGCAAGTACGAGAAACTGGAGGGCAAACAATTTTCTCTTTCTGATGCTGAGTATTTC
GTGTTCCATTCTCCATATAATAAGCTTGTACAGAAAAGCTTTTCTCGTTTGTTGTTTAAT
GACTTCACGAGGGATGCCAGTTCTGTTGACAAGGCTGCCAAAGAAAAGCTGGGACCA
TTTTCAACCTTATCTGGTGATGATAGCTACCAAAGCCGGGACCTAGAGAAGTTGTCCC
AACAACTTGCAAGGCCCCTTTATGATGCAAAGGTCCAACCATCCACTTTAATACCAAA
GCAAGTTGGTAACATGTATACAGCATCTCTCTATGCGGCATTTGCTTCCCTTGTTCACA
ACAAACAGTGTGAATTGGATGGCAAGCGAGTGGTTTTGTTCTCCTATGGAAGTGGTTC
AACTGCCACAATGTTCTCATTGAAACTTAATGAAGGTCAACATCCCTTCAGCTTGTCA
AACATCGTATCTGTCATGAATGTTGCTGGGAAGTTGAAGTCAAGGCATGAGTTTCCTCC
AGAGAAGTTTGTGGAAGTAATGAAGATAATGGAGCATAGGTATGGAGCCAAGGACTT
CGTGACGAGCAAAGGACAGTAG

HMGCR

ATGGACGTCAGCCGGCGGCCTACGAGAGCAGCACGTGTCGACGAGAACAAAGAGAA
GCGATCGACGTCACCGACGCCAAAAGCATCAGACGCGTTGCCGCTTCCGTTATACCTA
ACTAACGCCATTTTCTTCACGCTGTTCTTTTCCGTTGCGTTTTACTTGCTTCACCGTTGG
CGTGACAAGATCCGTAACTCAATTCCCCTCCACGTGGTCACTCTTTCCGAAATTGCCG
CCATTGTCTCGCTCATCGCTTCCTTCATCTACCTCCTTGGCTTCTTCGGCATTGATTTCGT
CCAGTCGTTCATCTCACGCGCCTCACCTGACGCGTGGGATCTCGATGACGATAACGAA
CAAATCAATGAAATCATCTCCCGTCCTACCGCTCCGATTTCCAAATTAGTCCCTCTCGA
TCCCATAATTACAACTCTGTCCTCCGTCGAAGACGAGGAAATCGTTAAATCCGTCATC

GACGGTGCGATTCCGTCTTACGCGCTCGAATCCAAGCTCGGCGATTGCAAACGAGCG
GCTGCGATTCGACGCGACGCATTGCAGAAAATGACTGGGAGGTCGTTGCAGGGATTG
CCGTTGGATGGATTCGATTACGAGTCGATTTTAGGTCAGTGCTGTGATATGCCGGTGGG
GTACGTGCAGATACCTGTGGGCATTGCGGGGCCGTTGTTGCTCGATGGGTTTGAATTCT
CGGTTCCCATGGCGACCACTGAAGGGTGTTTGTTCGCTAGTGCTAATAGAGGGTGTAA
AGCGATCTATGCTTCCGGTGGGGCCGCTAGTATCTTGTTGAGAGATGGGATGACGAGG
GCGCCTGTTGTTAGATTTGCTACTGCTAAGAGAGCTTCTGAATTGAAGTTCTTTTTGGA
GGATCCGAACAATTTCGATACTTTGGCCGTCGTTTTTAACAGGTCCAGTAGATTCGCTA
GGCTCCAACATATTCGATGTTCTATTGCTGGGAAGAATCTTTACATCAGATTTTGCTGTA
GCACTGGTGACGCAATGGGAATGAACATGATATCCAAAGGAGTTCAGAATGTTCTTGA
ATTCCTTCAAAATGATTTTCCAGACATGGATGTCGTTGGATTATCTGGTATGTCTCTTGT
CATTTTTATAGTTATATTTTTCTTCTTTCAATCGTTATTGAAATTTGGCTTTTTCCTCTAG

MVK

ATGGTGGTAGTTGCTTCAGCTCCTGGGAAGGTGTTAATGACTGGGGGTTACCTTATATT
GGAGAGACCCAATGCTGGGATTGTGTTGAGTACTAATGCTAGATTTTACGCCATTGTTA
AGCCTCTTTATGAACAACTCAAGCCTGACAGCTGGGCATGGGCTTGGTCTGATGTTAA
GTTAACATCGCCTCAGTTGTTGAGGGAAAGTATGTACAAGTTGTCGTTGACGAATTTAA
ATCTTCAGCCTGTATCTTCAAGTGAATCGCGGAACCCTTTTGTGGAATATGCAGTGCAA
TATGCCGTGGCAGCTGCACAGGCCGTATTTGATCAGAATAAAAAGGATGTGTTGCGCA
AACTACTCTTGCAGGGTCTTGATATCACAATTTTGGGATGCAATGACTTCTACTCATATC
GGAATCAGATTGAAGCACGTGGGCTTCCTTTGACACCAGAAGCATTGGCTGCCCTTCC
ACCCTTTACATCAATAACTTTCAATGCAGAGGAATCAAGTGGAGGAAATTGCAAGCCT
GAAGTTGCGAAAACTGGATTGGGTTCATCTGCAGCCATGACAACTGCTGTGGTTGCTG
CATTACTTCATTACCTTGGGGCTGTCAATCTTTCCGCTTTCAGCTGA

MVAK

ATGGCAATCTCCGTCTCTCTAGTTTCATCATCAAATTTGTTATCATTTTCATTATCTTCTA
CTTTGGGTCCTTTTAGAAGCCCTCAAATTAGATTCATGAGTTTTCATAATCAGCATCAG
AACTCAAATGGACGGTTGGTGATCAACTGTTGCTCTTCTTCTGACATGGGTTCAACTCA
AAGCCCCATGAATATGAGGCAACCTCAAATGTCATCTATGTCTCCCTTTGGAGTGACAT
TGAATGACAACGGCATGTCAAAGTCATCCTACAAATGGCAAAGGGTGTTGCTTAAAGT
GAGTGGAGAAGCACTGGCTGGAGATCACACAAAAAACATTGACCCAAAGGTTACGA
TGGCCATTGCTAGAGAGGTTGCATCAGTTACTCGCCTTGGCATAGAGGTTGCTATTGTG
GTTGGTGGGGGAAACATATTCCGCGGGGCCTCAGCAGCAGGAAATAGTGGCCTTGAC
CGCTCATCCGCTGATTATATTGGGATGTTAGCGACTGTCATGAATGCTATATTTCTTCAA
TCAACAATGGAGAGTATTGGCATTCCCACCAGGGTGCAGACTGCTTTTCGTATGTCAG
AAGTAGCAGAGCCATATATACGGCGAAGAGCTGTGAGGCATTTGGAGAAGGGTAGGG
TTGTGATTTTTGCAGCTGGAACAGGAAATCCATTCTTCACCACAGATACTGCTGCAGC
ACTTCGATGTGCTGAAATTAATGCTGAGGTTGTTTTGAAAGCTACAAATGTTGATGGGG
TCTATGATGATGATCCTAGACGTAACTCAAATGCTCGTGTTCTTGATACTCTAACATATC
ATGAGGTGACTTCAAGACATCTTTCGGTAATGGACATGACTGCAATTACTTTGTGCCAA
GAAAACAACATTCCAGTTGTGGTCTTCAACCTAAACCATCCCGGTAACATCTCAAAGG
CCATAAAGGGAGAGAGGGTTGGTACATTGATTGGAGGGTCATGGAATTCAACAGTGTC
AAAGACATGA

MVD

ATGGCGGCGGAGAAATGGGTGATGATGGTGACGGGACAGACCCCGACGAACATAGC
GGTGATTAAGTACTGGGGGAAGAGAGACGAAACCCTAATTTTACCTGTCAATGATAGT
ATTAGCGTCACTCTTGATCCCGATCACCTCTGTACTACCACTACTGTTGCTGTCAGTCC
AAGCTTTGATGAAGATCGTATGTGGCTCAATGGAAAGGAAATTTCGCTTTCTGGGGGA
AGGTACCAGAATTGTTTGAGGGAAATCCGGAGTCGGGCTTGTGATGTTGAGGAAAAG
GAGAAAGGGATCAAGATTGAGAAGAAGGATTGGGAGAAATTACATTTACACATTGCT
TCATATAACAATTTTCCCACGGCAGCTGGGTTGGCTTCCTCTGCTGCCGGTTTTGCCTG
CCTTGTTTTTTCCCTGGCTAAGCTGATGAATTTGAAAGAAAATCAGAGCCAGCTTTCTG
CTATAGCTAGGCAAGGTTCAGGGAGTGCTTGTCGTAGTCTTTTTGGTGGATTCGTCAAG
TGGATAATGGGAAAAGAAGAAAATGGAAGTGACAGCCTTGCTGTTCAACTTGTAGAT
GAAGAGCACTGGAATGATCTGGTTATTATTATTGCTGTGGTGAGTTCACGGCAGAAGG
AAACAAGTAGTACCTCAGGAATGCGTGAGAGTGTTGAAACAAGTTTGCTTTTACAGC
ATAGAGCAAAGGAAGTCGTACCAAAACGCATAATACAAATGGAAGAAGCCATCCAA
AATTGTGATTTTTCGTCTTTTGCACAATTGACCTGTGCTGATAGTAATCAATTTCATGCT
GTTTGCTTGGATACCTCTCCCCCTATATTTTACATGATTGATACATCCCACAGGATTATC
AGCTATGTTGAAAAATGGAATCGTTCCGAGGGATCACCTCAGGTGGCATATACTTTTG
ATGCTGGCCCGAATGCTGTTCTGATTGCTCGTAATAGAAAGGTTGCTGCCCAATTGCTT
CAAAGGCTACTTTTCTTTTTCCCCCCGTCTGATACAGAATTGAACAGTTACGTTCTTGGT
GATAAGTCAATCCTACAAGATGCCGGCCTTGAGGGGATGAAAGATGTGGAAGCGTTA
CCACCACCTCCGGAAATTAACAACATAGCAGCCCAGAAGTATACAGGGGATGTCAAT
TATTTTATCTGTACAAGACCAGGAAGAGGTCCTGTTTTGCTTTCTGATGACAGTAAGGC
TCTTCTCAACCCTGAAACTGGTCTGCCTAAGAAGGCTTAG

GPP/FPP

ATGGCTTCAGCTCTCATGAGCAGCTCCATCAATGCTCTCCAACACCACTCAAAAATCA
CATTATGCGACCCTAAACTTGTCCCAAAGTCAGCCCATGTTCCATATTCATCATTTAAG
CAACCAATCATATCCAAAACCCTGACGATCAAGTCAAGAAACAAACCCCCCTTGCCA
GGATTCCAAATTGATGAATACATGACAACAAAAGCAAAGAAAATCAATAAGGCACTA
GATGAAGCAATTCCATCAATATTGCAAAATCCAACGCTACTCGATCACAAAAGGGAA
CGTGTGTATGGAACTCTATGCATTGCTTCATGTGAGTTGGTAGGAGGCAATGAATCGTT
AGTGATGCCACTGGCATGTGCTACAGAAATGTTCATGACAATGGTTCTTAATCGTACTC
TTCTCTGCCATGCAATTGAGTATGTGGCAACGAAGACCAACGGAGTTTCGCCTGACCA
GCTAGTGAAAGCCATCGGCGAAATCACGTCAGCAGTAGGCACAAGAGGAGTCTTGGC
AGGGCGGATCATGGACATTAAAAGCAAGGGAAAAGAATTGAGTTTGAGTGAATTGGA
GATCATTCAGAGGCTCAAAATTGGCAAGTTTATTGAGGCTTCGGTTGTGTCAGGAGTTT
TAATCGGAGGGGGAAATGAAGAAGAGATGGAGAGGCTGAGGAAGTATGGAAAATGT
GTAGGCTTAGCGTATCAAGTGTGGGATGATGTTTTAGGTGTGGGGATGGAGGCTGACG
ATAAGGCAACGTACCCAAAGTTGATGGGTGTGGATGGAGCTAAGAAGTACGCATGGG
AGCTGGTGGCTGAAGCAATTGAAGTGCTTGCTTATTTTGACTCTACGAGGGCTGACCC
CTTGTATCATTTGGCTAATTTTGCGGTTTCTCGACGAATTTAA

IDI

ATGGGTGACGCTACTCCTGATGCTACCATGGACGCTGTCCAGCGACGCCTCATGTTCG
AGGACGAATGCATTTTGGTGGATGAGAATGACCGTGTTGTTGGTCATGAAAACAAATA
CAACTGTCACTTGATGGAAAAGATTGAATCTTTGAATTTGCAACACAGAGCTTTAAGT
GTATTTTTGTTTAACTCCAAATATGAGCTGCTCCTTCAGCAACGCTCTGCAACAAAAGT

TACATTCCCTTTTGTGTGGACTATCACCTGCTGCAGCCATCCTCTATTCCGCGAATCTGA
GCTTATTGAGGAGAATGCTCTTGGGGTGAGGAATGCTGCACAAAGAAAGCTTTTGGAT
GAGCTGGGTATTCCTGCTGAAGATGTGCGGGTTGATGAGTTCACTCCACTGGGTCGCA
GTCTGTACAAGGCACCTTCTGATGGCAAGTGGGGGGAGCATGAACTTGATTACTTGCT
CTTCATTGTCCGAGATGTTAATGTTAACCCAACACCCTGA

SQS

ATGGGGAGTTTGGGAGCGGTGTTGAAGCACCCAGATGATGTTTACCCGCTATTGAAGC
TGAAAATGGCGGCGAGGGAAGCTGAAAAGCAGATCCCATCTGAGCCTCACTGGGCCT
TTTGTTACTCTATGCTTCATAAGGTATCGAGAAGTTTTGCTCTTGTTATTCAGCAGCTCG
GTACTGACCTCAGAAACGCTGTTTGCATATTTTATTTGGTTCTTCGAGCCCTGGACACT
GTTGAGGATGACACCAGCATACCTACAGATGTCAAAGTGCCTACTCTGATAGCTATTC
ATCGTCATGTGTATGACCATGAATGGCATTTTTCATGTGGTAAAAAGGATTACAAAGTG
CTCATGGATCAATTTCATCATGTTTCAATTGCTTTTCTGGAGCTTGGAAAAGGTTATCAG
GAGGCGATTGAGGATATTACCAAAAGAATGGGTGTTGGGATGGCAAAATTTATTTGCA
AAGAGGTAGAATCAATTGACGACTATTATGAATATTGCCACTATGTAGCAGGACTTGTT
GGATCAGGTCTGCCAAACTTTTCCATGCCTCTGGGAAGGAAGATCTGGCTCCAGATAA
TCTCTCGAATTCGATGGGTTTATTTCTTCAGAAAACAAACATCATTCGAGATTATCTGG
AAGATATTAATGAGATACCAAAGTCGCGCATGTTTTGGCCTCGTGAGATCTGGAGTAA
ATATGTTATTAAACTTGTGGACTTAA

SQE

ATGGCAGATCAGTACACGTGGGGATGTATATTGGCCGCAGTGCTGGGGTTAGTGGCCG
CGTATAATCTCGGCCTTCTTGCTCTTTATAATCTTGTTATGAAGAAGAAGAAGAAGAGC
AACAACAACACTACTACAGATTCCTTCCACAAGACCGCCGCTACTCCTGTTCTAAATG
GAGAATACCTCTTCCATGCTGACGTCATCGTCGTCGGTGCCGGCGTCGCTGGCGCCGC
TCTCGCTCACACTCTCGCCAAGAATGGACTTCGAGTGCGTGTGATAGAAAGAGATTTG
TCGGAGCCTGACCGAATTGTTGGTGAATTGCTGCAACCAGGGGGCTACCTAAAATTAA
TTGAGTTAGGACTTGAAGATTGTGTGGAGGAAATTGATGCTCAACTGGTTTTTGGTTAT
GCACTTTTCAAGGATGGAAATAATGCCAGACTATCATATCCCTTGGAAAAGTTTCACTC
AGATGTTGCAGGGAGGGGATTTCATATTGGACGTTTCATACAGAGGCCGCGGGCGAA
AGCGGCTTCCCTTCCAAATGTACGATTGGAGCAAGGAACTGTCACTTCTCTGCTTGAA
GAAAAAGGGACTATTAAAGGTGTGCAATACAAGACTAAAGCTGGTGAAGAACTGAC
AGCATATGCTCCTTTGACCATCGTGTGTGATGGCTGTTTTTCAAACTTGCGTCGCTCACT
TTGCAATCCTAAGGTAGATGTGCCGTCCTGTTTCGTTGGCTTGGTCCTTGAGAATTGCA
ATCTTCCATTTGCAAATCATGGGCATGTTGTACTAGCGGACCCTTCCCCCATTTTGTTTT
ATCCAATCAGTCGTAACGAGGTTCGGTGTCTGGTTGATATACCTGGTCAGAAGGTCCCT
TCCATTTCAAATGGTGATCTGGCAAACTATTTGAAGACAGTAGTTGCTCCTCAGATTCC
ACCTCAAATCTTTGATTCCTTTGTTGCTGCTGTTGATGGAGGAAATATTAAAACAATGC
CAAATAGAAGCTTGCCAGCTGCTCCCCATTCCACTCCTGGAGCTCTTTTGATGGGGGAT
GCATTCAACATGCGGCATCCATTAACTGGGGGAGGAATGACTGTGGCACTGTCTGACA
TTGTCATCCTGAGTAACCTTCTTAGGCATTTGCGTAACCTGAATGATGCACCAGCTCTC
TGCAAATACCTTGAATCCTTTTACACCTTGCGTAAGCCAGTGGCATCCACTATCAATAC
ATTGGCAGGTGCCTTGTACCAGGTGTTTTCTGCTTCTCCTGACGAAGCAAGGAAGGAA
ATGCGTCAGGCTTGCTTTGACTATCTAAGTCTTGGAGGTATATTTTTCAGGACCCATCT
CTCTGCTCTCGGGCTTAA

CYP

ATGAGAACTGACATAGAAAGCCTCTGGGTCTTTGCTCTTGCTTCCAAATGCAGAGCCT
TCACTCAAGAAAACGTTGCATGGTCGCTCTTGATCATGTTTTTAGCTTGGCTAGCCATG
GCCCTTGTCTACTGGGCTCACCCTGGCGGTCCAGCTTGGGGCAAGTATAGGTTCAAAA
GAGGCTCATCTTTTGTTAAAGCCAACCCAATTCCTGGCCCTAGAGGATTGCCTGTAATT
GGCAGCATGAGCCTCATGGTGTCTCTGGCACACCACAGGATTGCAGCTGTAGCTGAAT
CATGCAAGGCTAAGCGGCTCATGGCCTTTAGCATGGGTGATACTCGTGTTATAGTAACG
TGTAATCCCGAAGTCGCAAAAGAAATTCTAAACAATTCTGTATTTGCTGATCGTCCAGT
TAAGGAGTCAGCTTACAGTTTGATGTTCAACAGAGCAATTGGTTTCGCACCTTATGGTG
TTTACTGGCGGACCCTGAGGAGAATCGCGGCCACTCATCTTTTTTGCCCGAAGCAAAT
CAAAGGAGCTGAGCCACAAAGATTAGTTATCGCATCTCAGATGGTGACCATGTTTGGG
GACCATAAAAAAGGTTTTTGCGTACGTGAAGTACTTAAACAAGCTTCACTAAACAATA
TGATGTGTTCAGTTTTTGGACGTGAATATAAATTAGATTCGTTCAATAACGAAGTTAAA
GAGCTAAGAGAATTAGTTGATGAAGGGTACGAGCTACTAGGCATGCTAAATTGGACCG
ATCACCTTCCTTGGCTAGCAGATTTTGACCCTCAAAAAATCCGGTTTAGATGTGCAACA
CTCGTACCAAAAGTGAACCGGTTCGTTAGTCGAATTATAGCCGAACACAGAGCTCAA
ACTGACCAAAAGAATCTGGATTTTGTCGACGTTTTGCTCTCTCTTCAAGGCTCAGATAA
GTTATCAGACTCCGATATGATCGCCGTCCTTTGGGAAATGATATTTAGAGGAACTGATA
CAGTGGCGGTTTTGATCGAGTGGATACTAGCAAGGATGGTGCTGCATACTGATGTCCA
ATCAAGGGTGCTAGATGAACTTGACAAGGTTGTAGGCAGATCACGCGCGGTGGTGGA
GTCTGATCTTGGGGAATTGATCTATCTAACGGCTGTGATTAAAGAAGTTTTAAGGTTAC
ATCCACCTGGGCCACTTCTCTCGTGGGCCCGTTTAGCCATATCTGATGCAACCATCGAT
GGGTATCACGTGCCTGAAGGGACCACTGCAATGGTGAATATGTGGGCCATTTCTAGGG
ACCCACAAGTGTGGAAGGACCCTCTTGAATTTATACCCGAGAGATTTGTGGCTAAAGA
GGGTGAGGTGGAATTCTCTGTTCTCGGATCGGATCTCAGGCTGGCTCCGTTTGGGTCA
GGTAGACGGGTTTGTCCGGGAAAGAACTTGGGCTTAACTGCGGTTAGCTTCTGGGTTG
CATCACTGTTGCACGAGTATGAATGGCTACCGTCGGATGAGAATGTCGTTGACCTATCT
GAAGTATTGAGGCTGTCTTGTGAAATGGCCAATCCTTTAACGGTTAAAGTGCGCCCAC
GGCGTAGGTTAAACCTATGTCATTAA

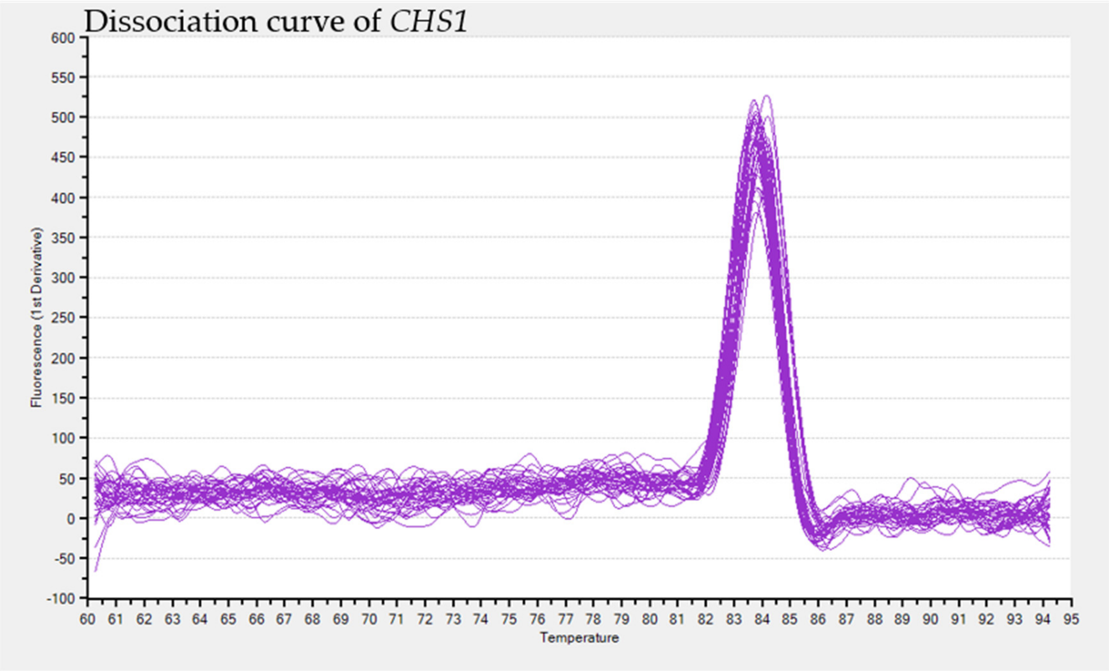

Figure S1. Dissociation curve of *CHS1*.

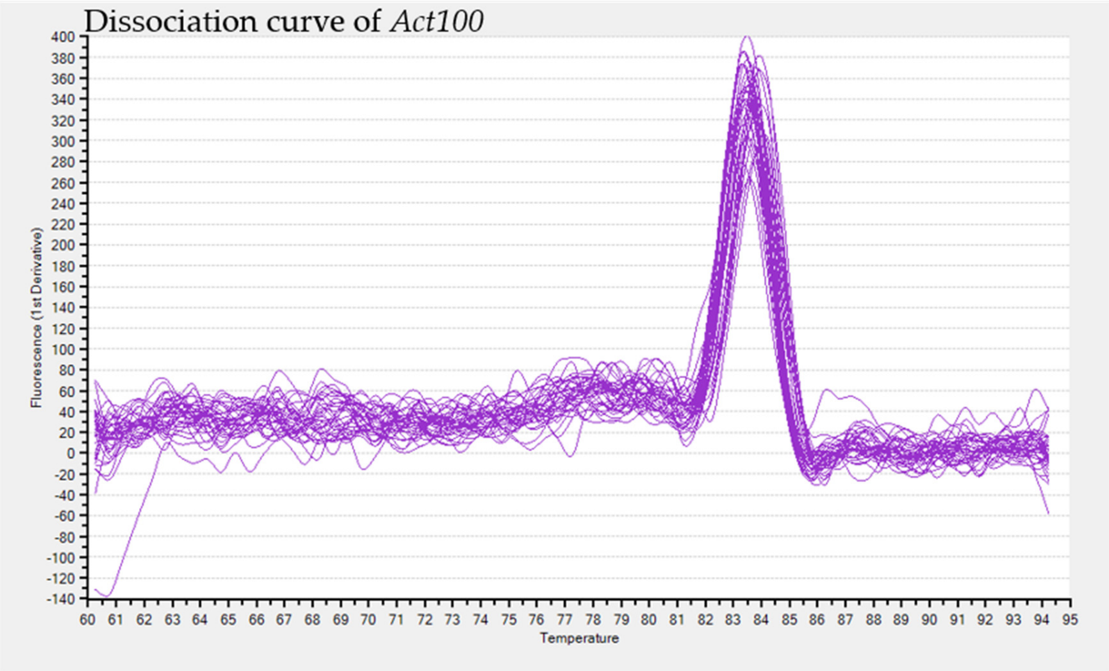

Figure S2. Dissociation curve of *Act100*.

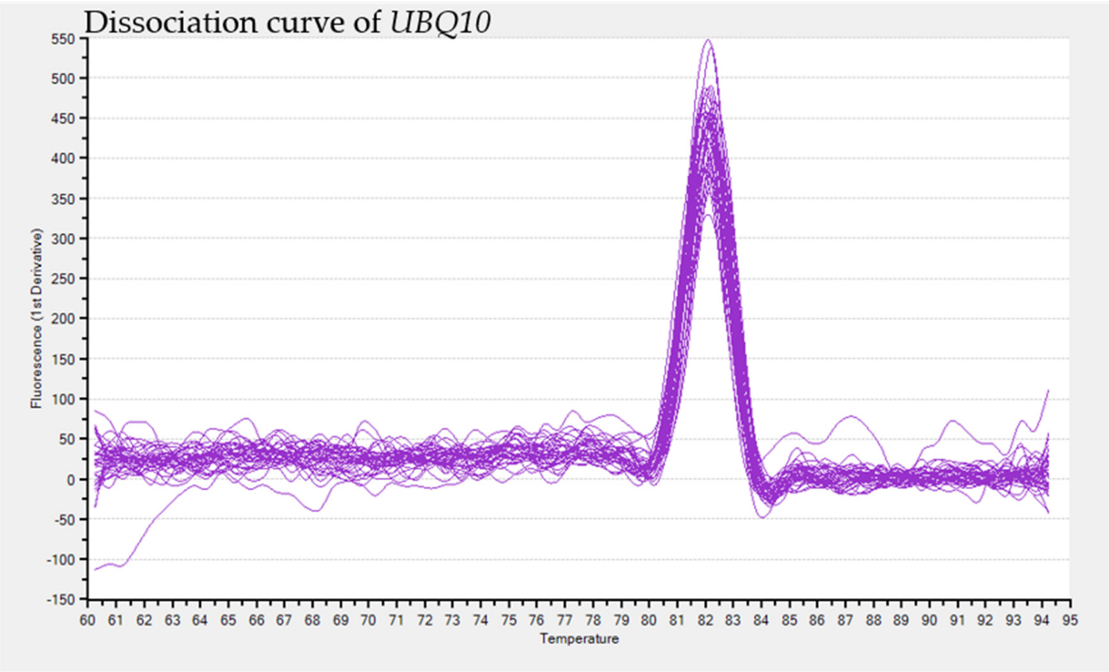

Figure S3. Dissociation curve of *UBQ10*.

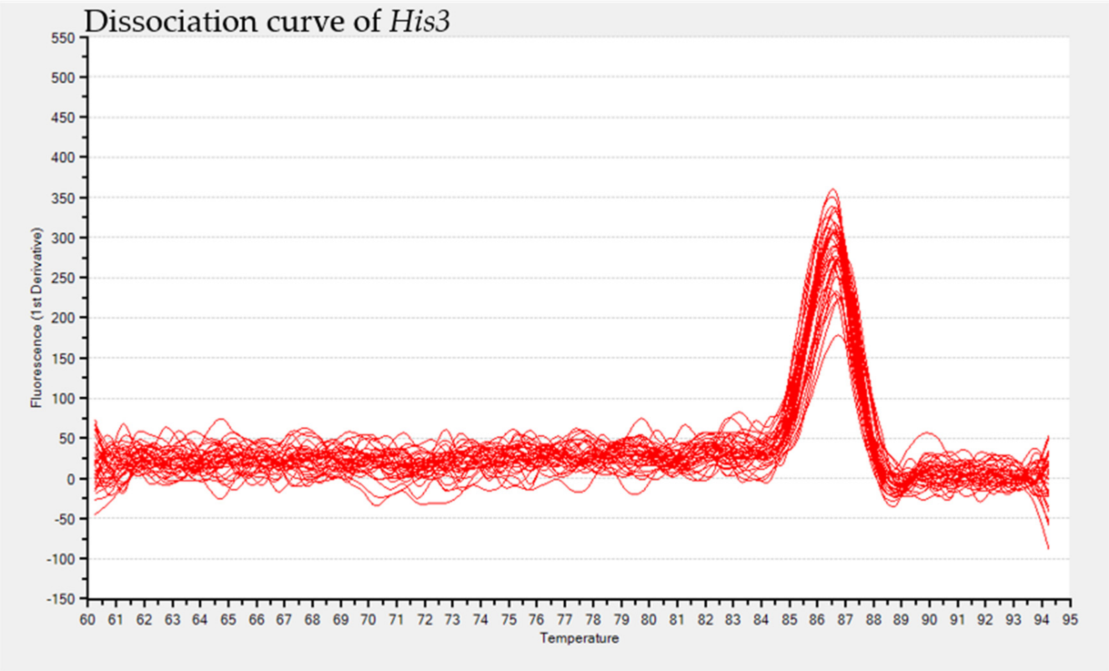

Figure S4. Dissociation curve of *His3*.

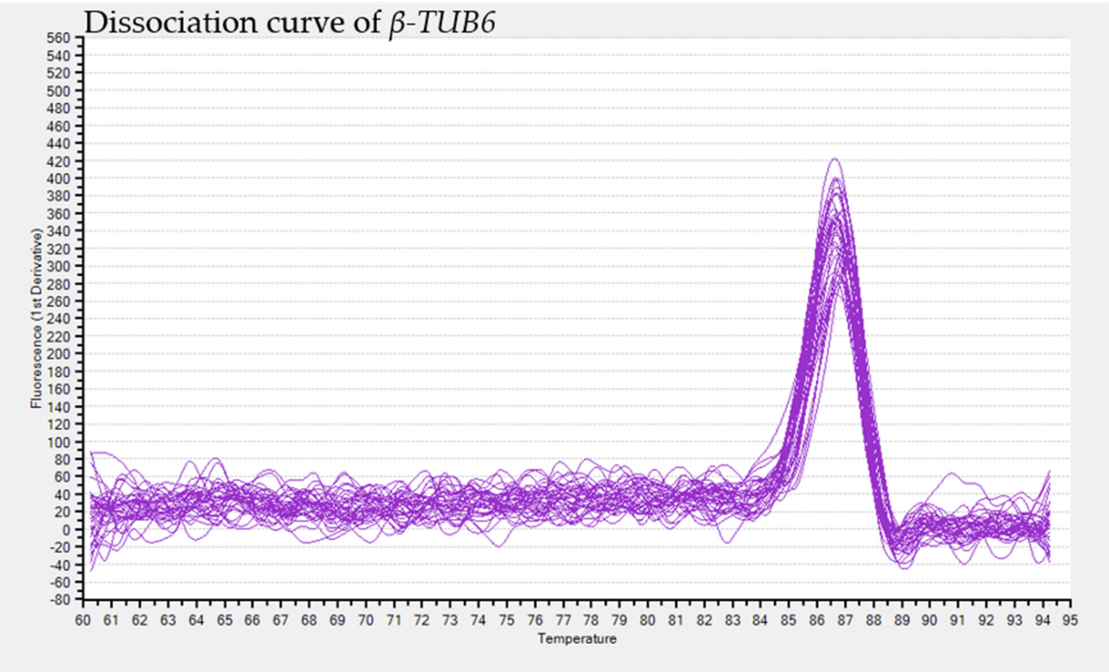

Figure S5. Dissociation curve of *β-TUB6.*

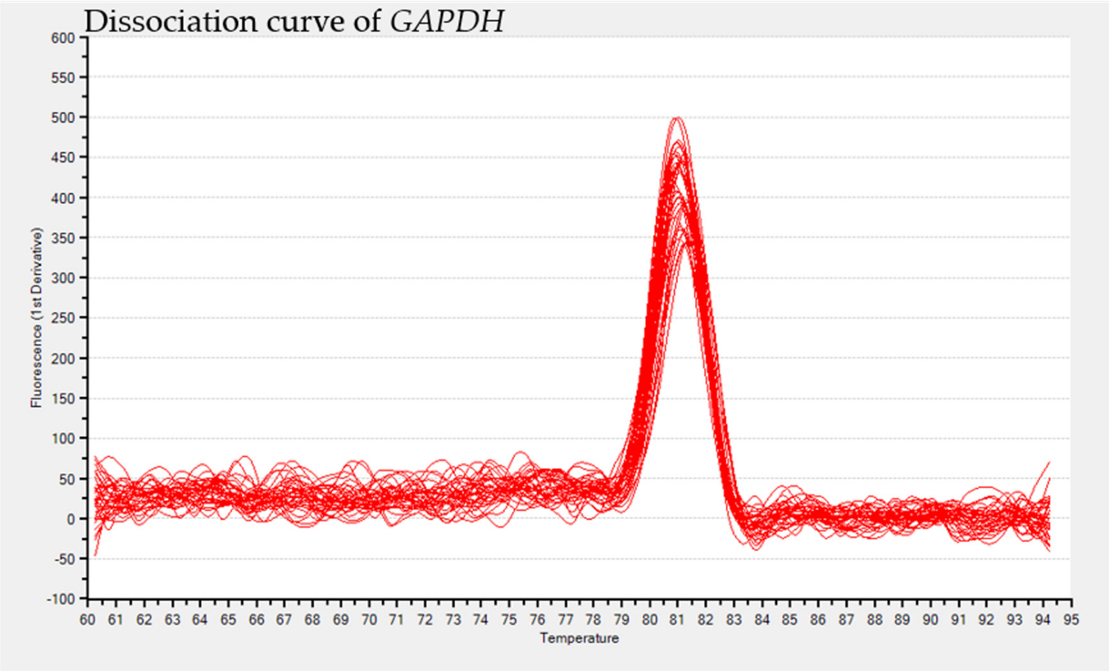

Figure S6. Dissociation curve of *GAPDH*.

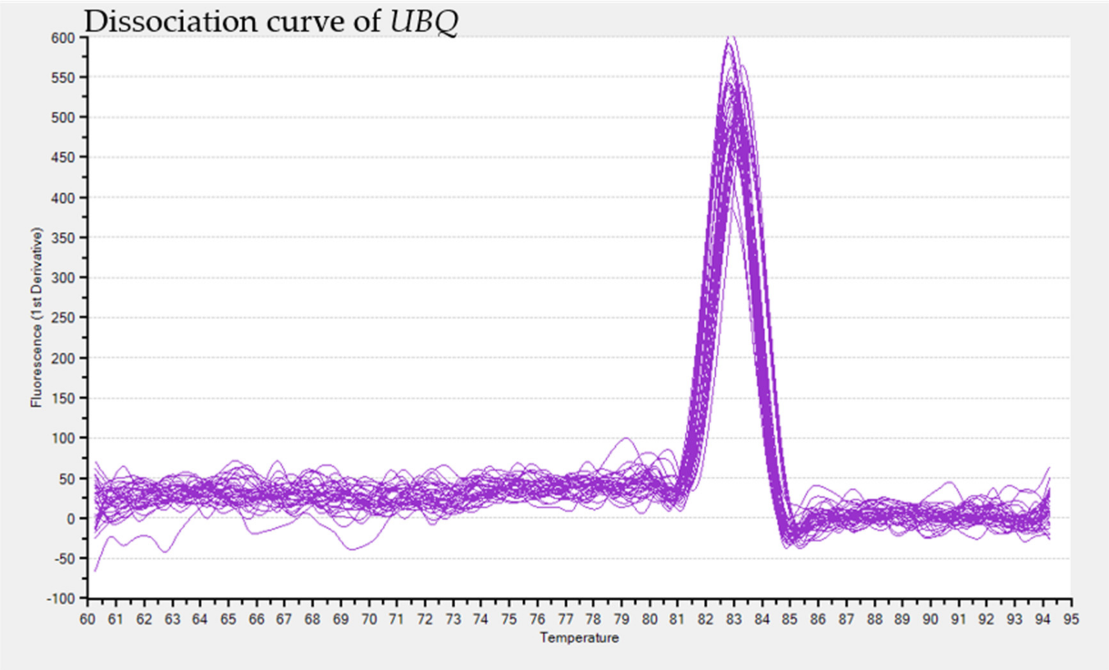

Figure S7. Dissociation curve of *UBQ*.

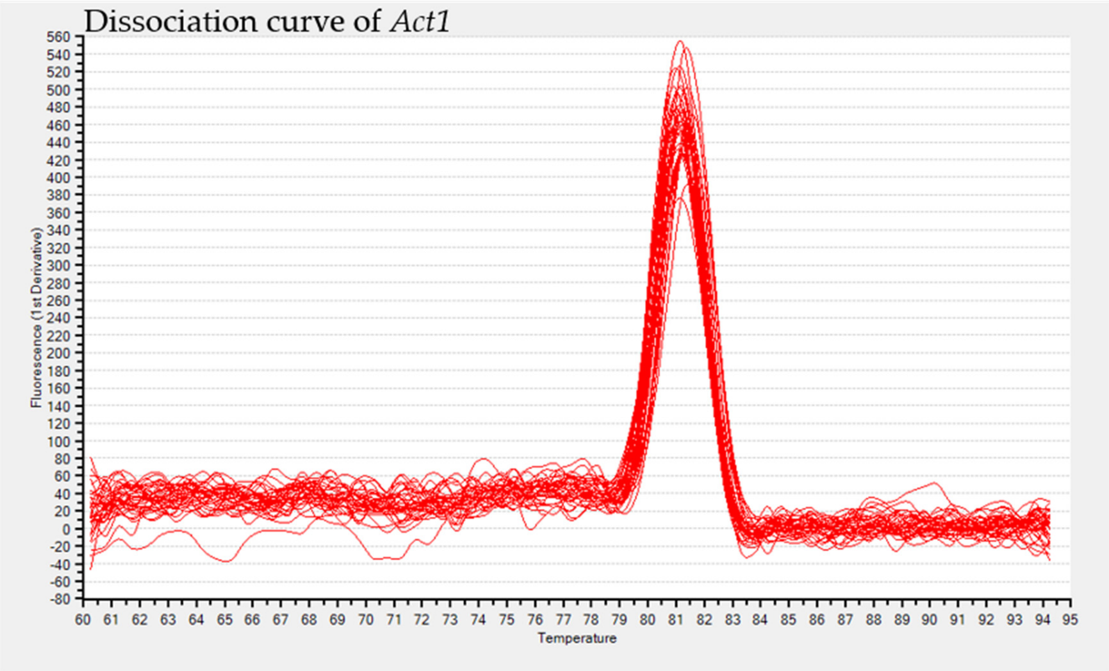

Figure S8. Dissociation curve of *Act1*.

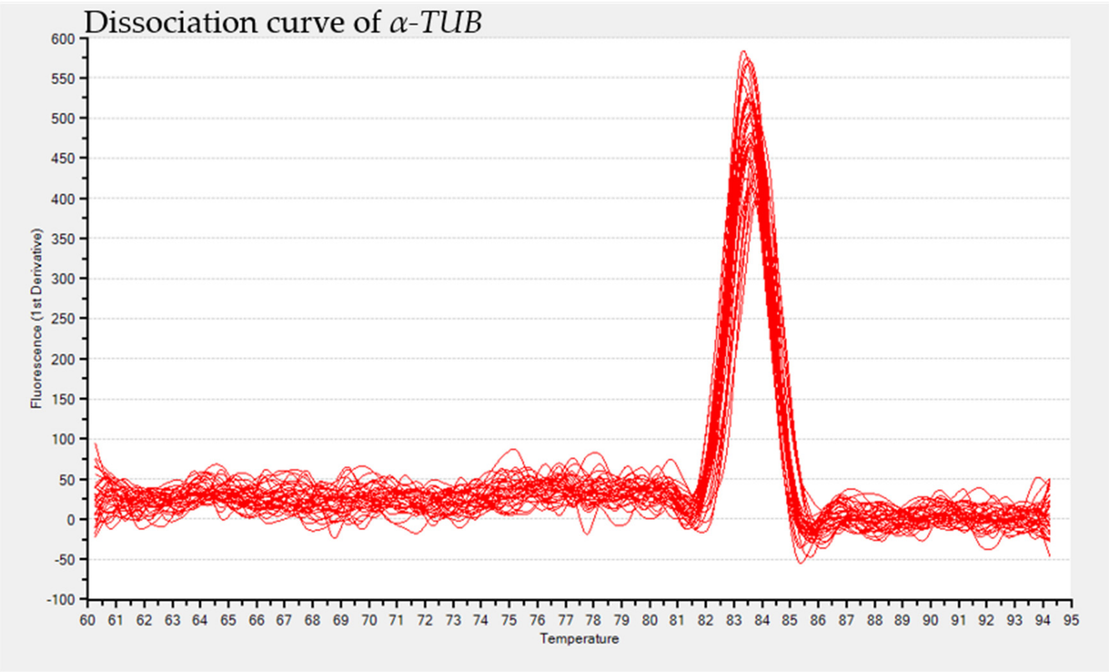

Figure S9. Dissociation curve of *α-TUB*.

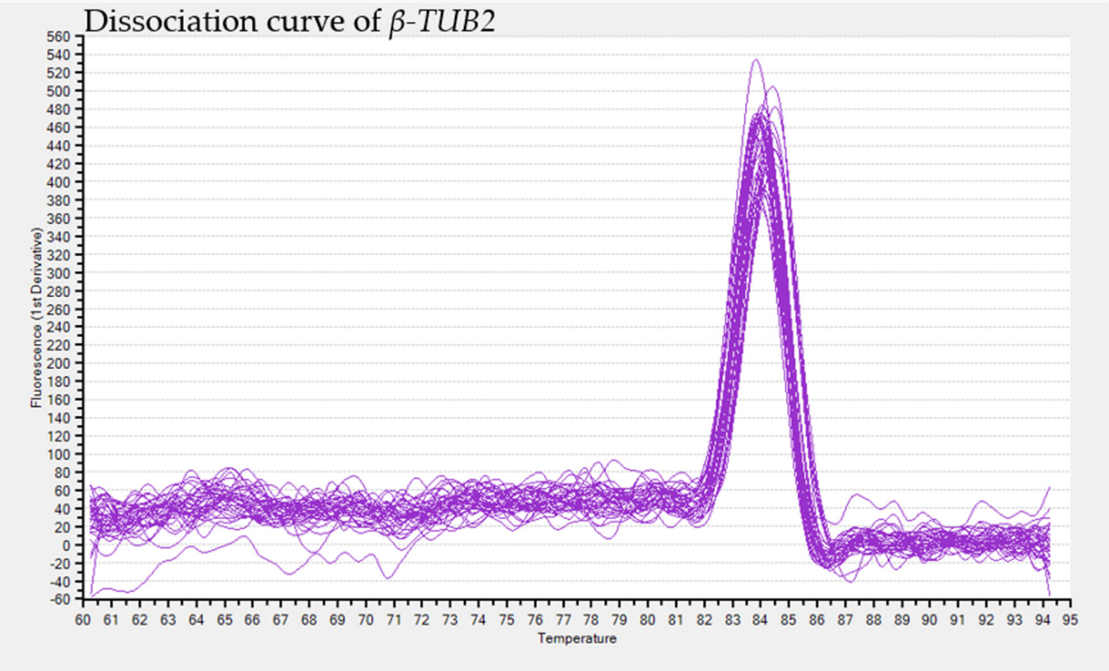

Figure S10. Dissociation curve of *β-TUB2*.

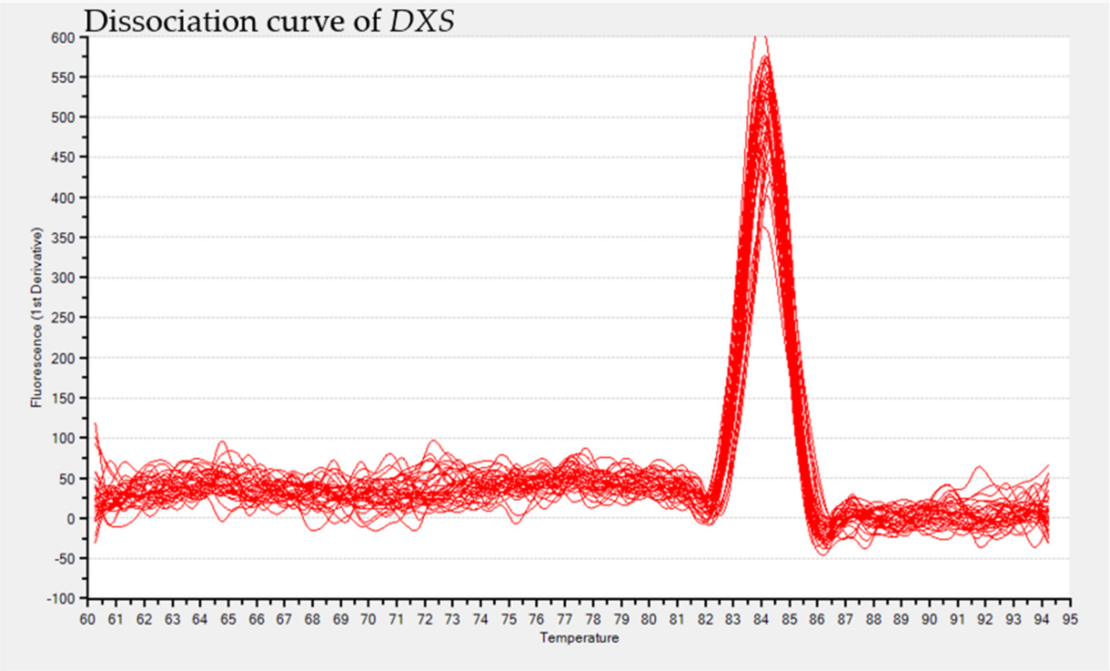

Figure S11. Dissociation curve of *DXS*.

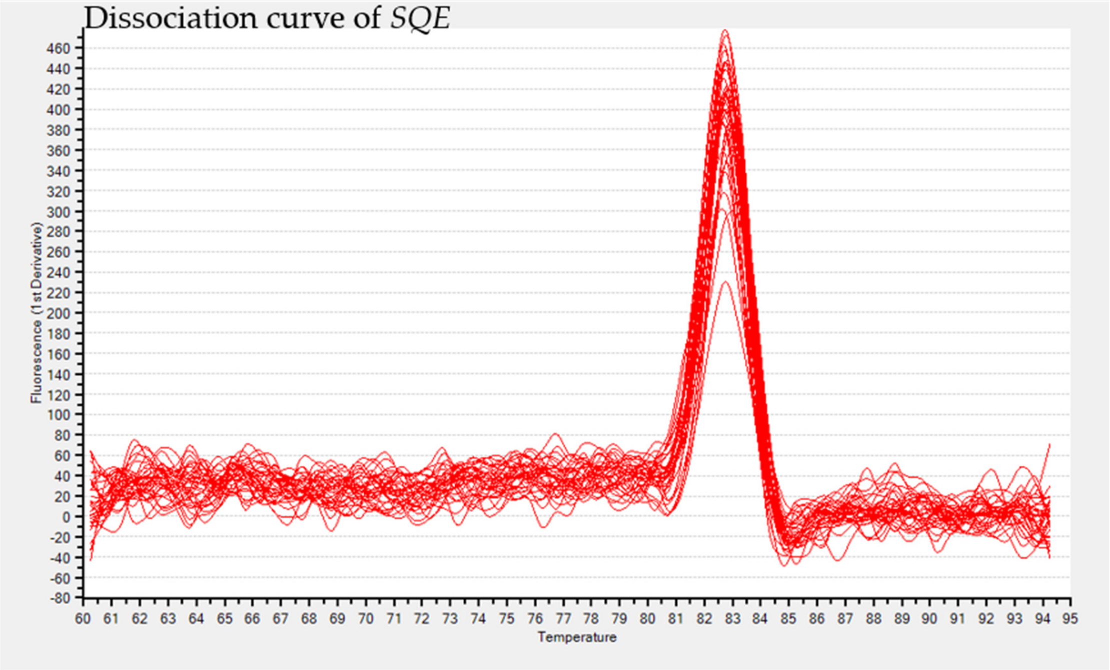

Figure S12. Dissociation curve of *SQE*.

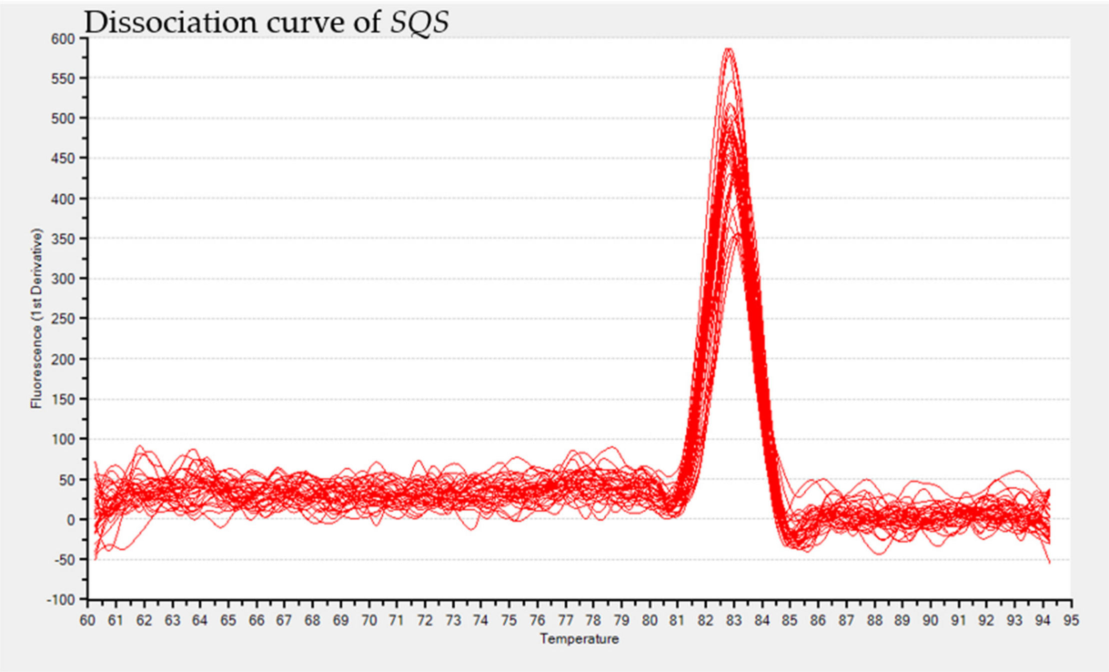

Figure S13. Dissociation curve of *SQS*.

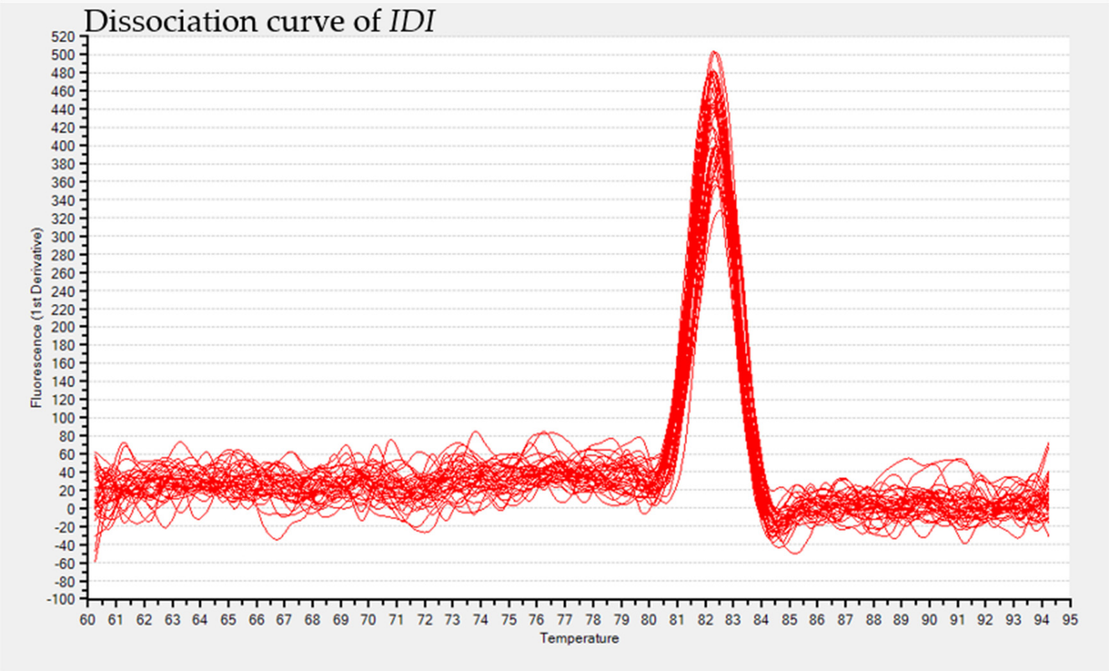

Figure S14. Dissociation curve of *IDI*.

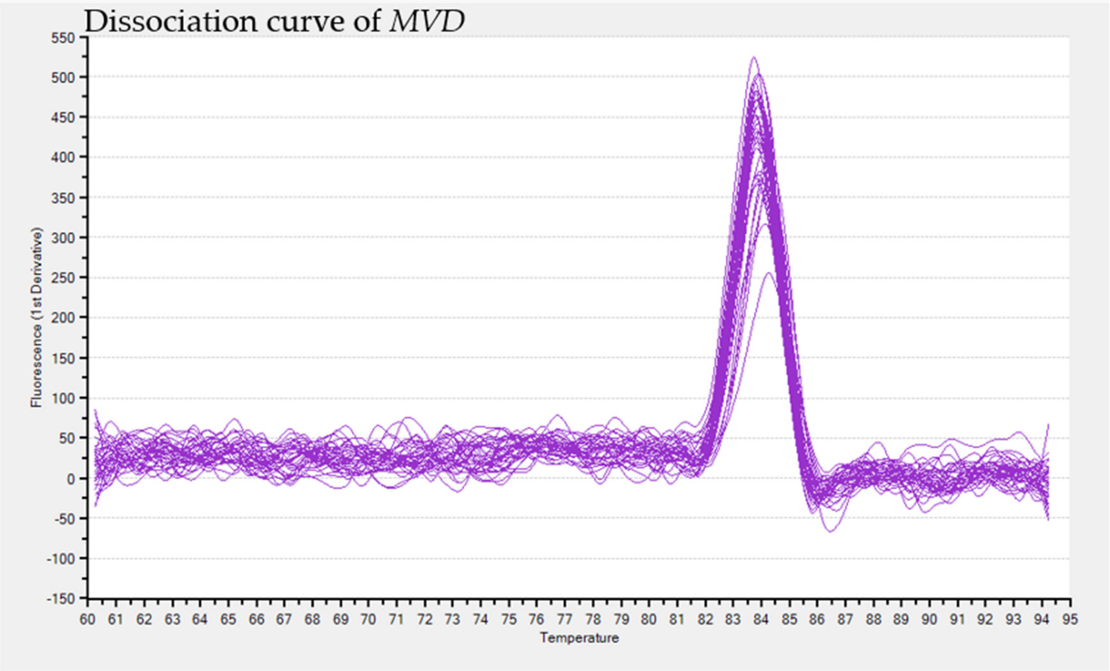

Figure S15. Dissociation curve of *MVD*.

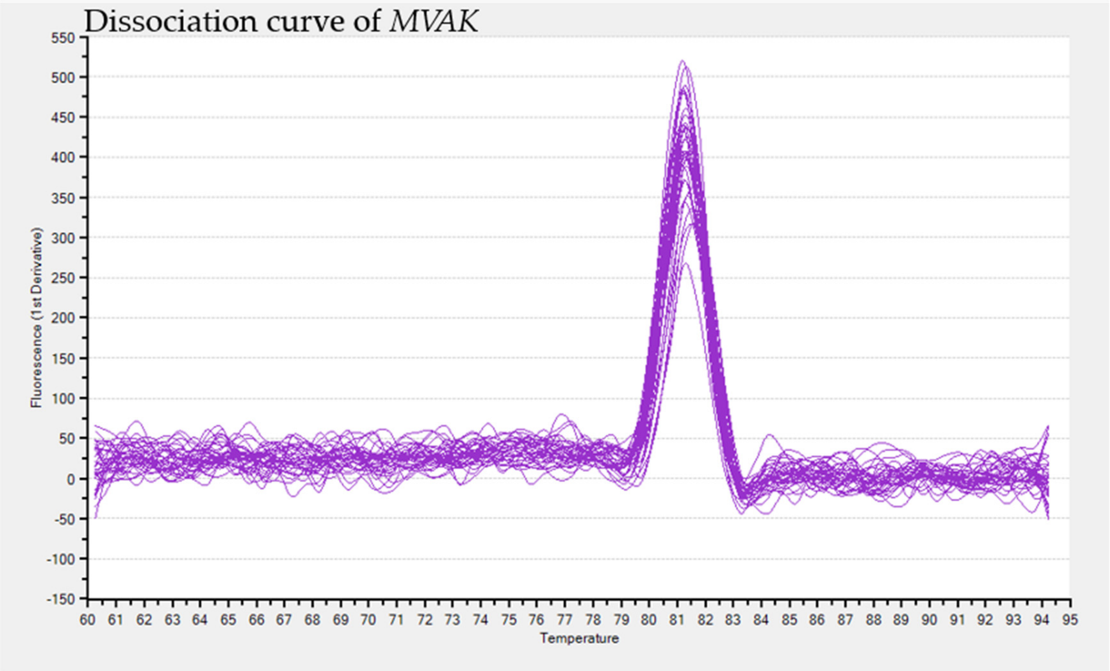

Figure S16. Dissociation curve of *MVAK*.

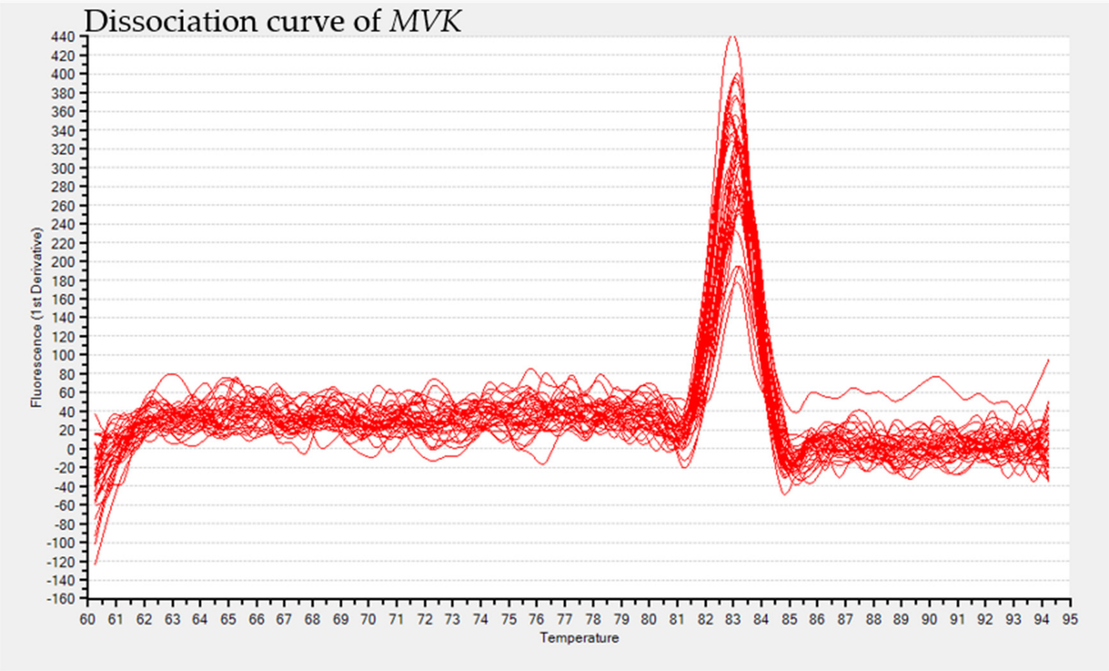

Figure S17. Dissociation curve of *MVK*.

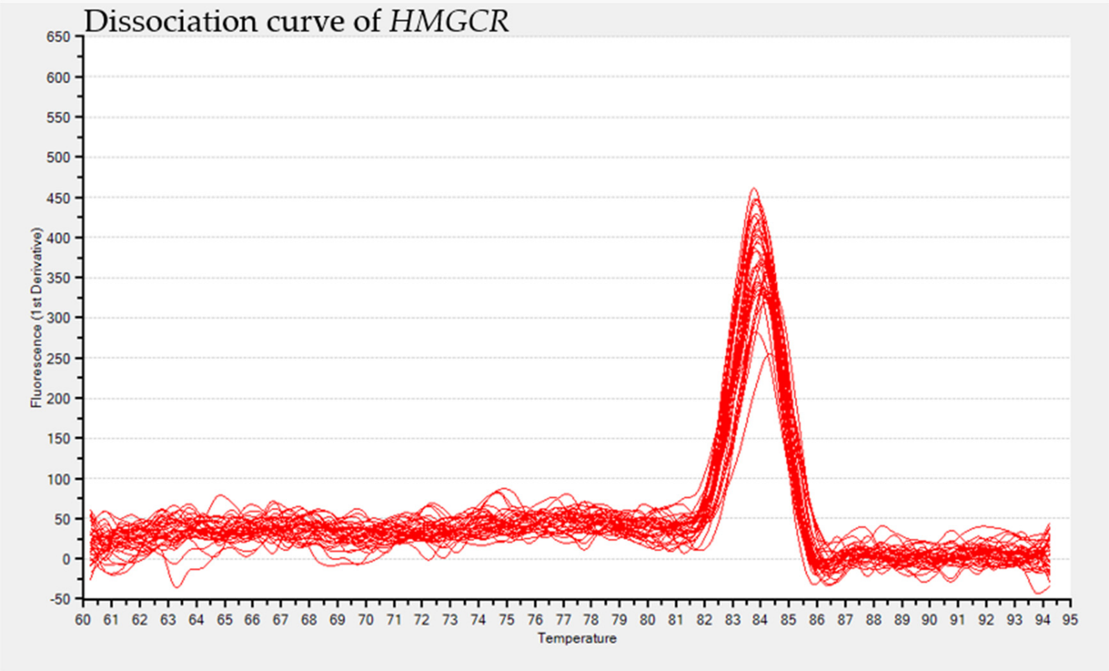

Figure S18. Dissociation curve of *HMGCR*.

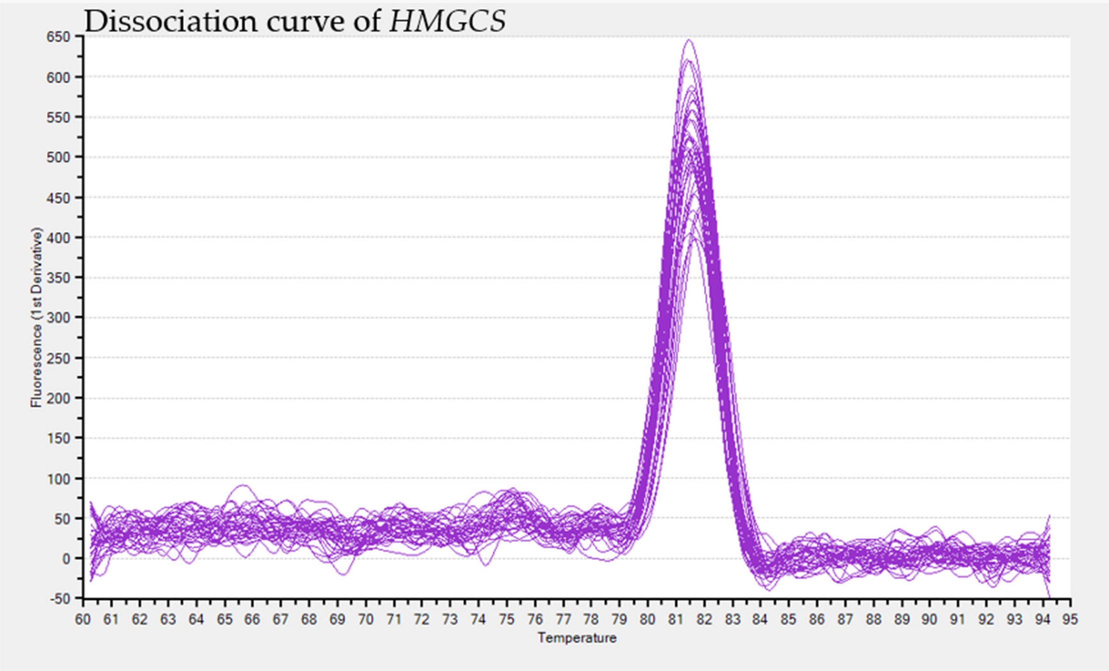

Figure S19. Dissociation curve of *HMGCS*.

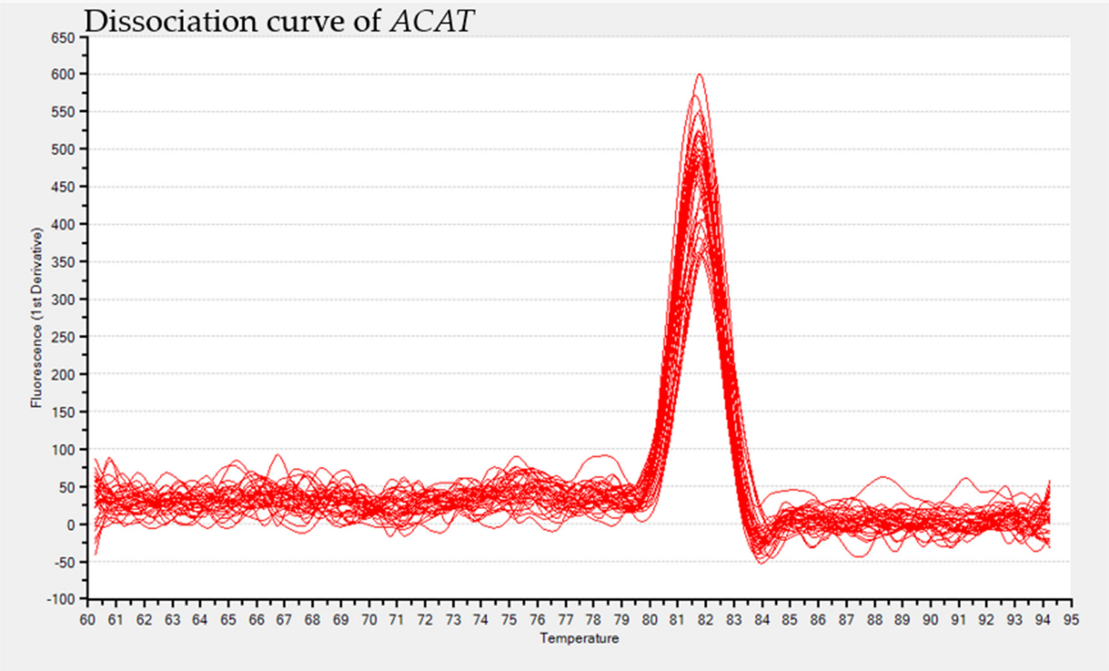

Figure S20. Dissociation curve of *ACAT*.

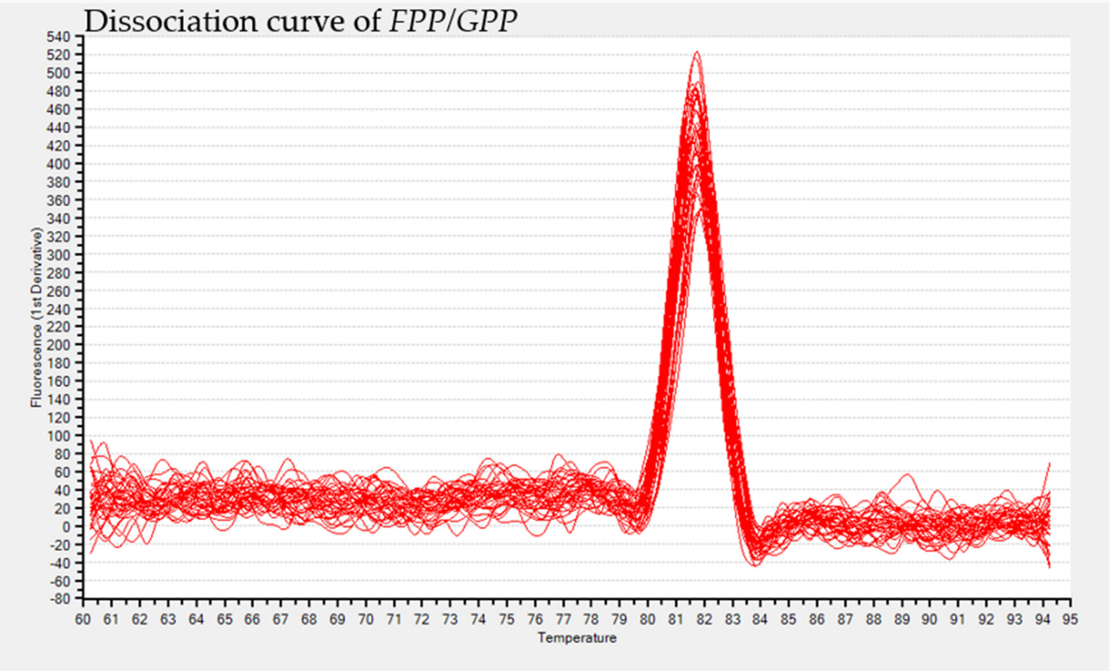

Figure S21. Dissociation curve of *FPP/GPP*.

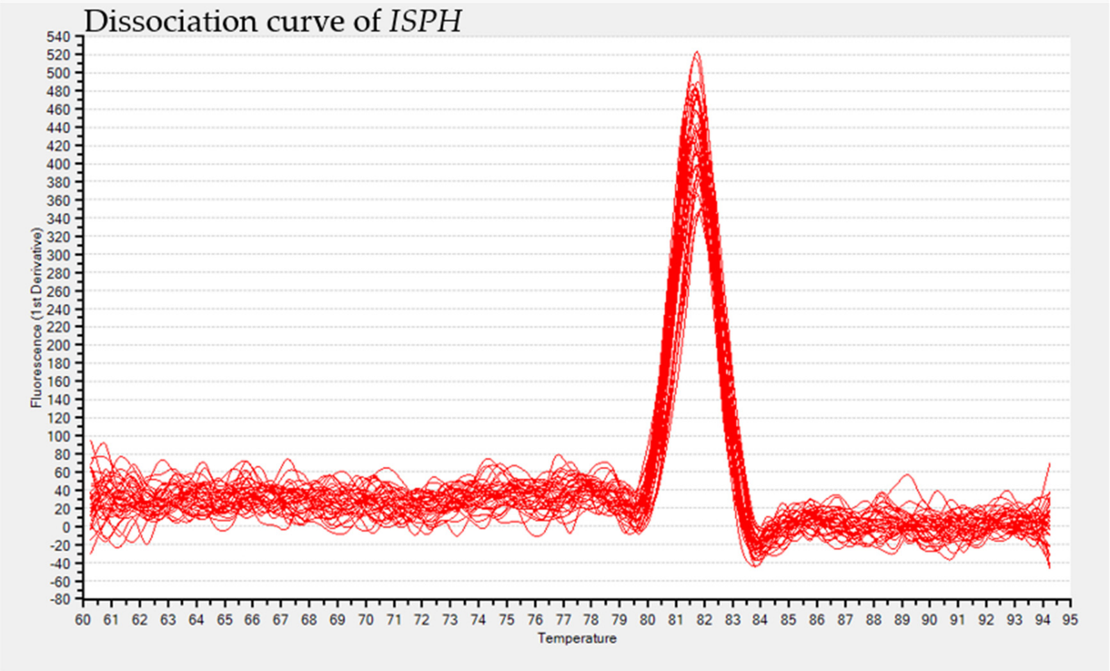

Figure S22. Dissociation curve of *ISPH*.

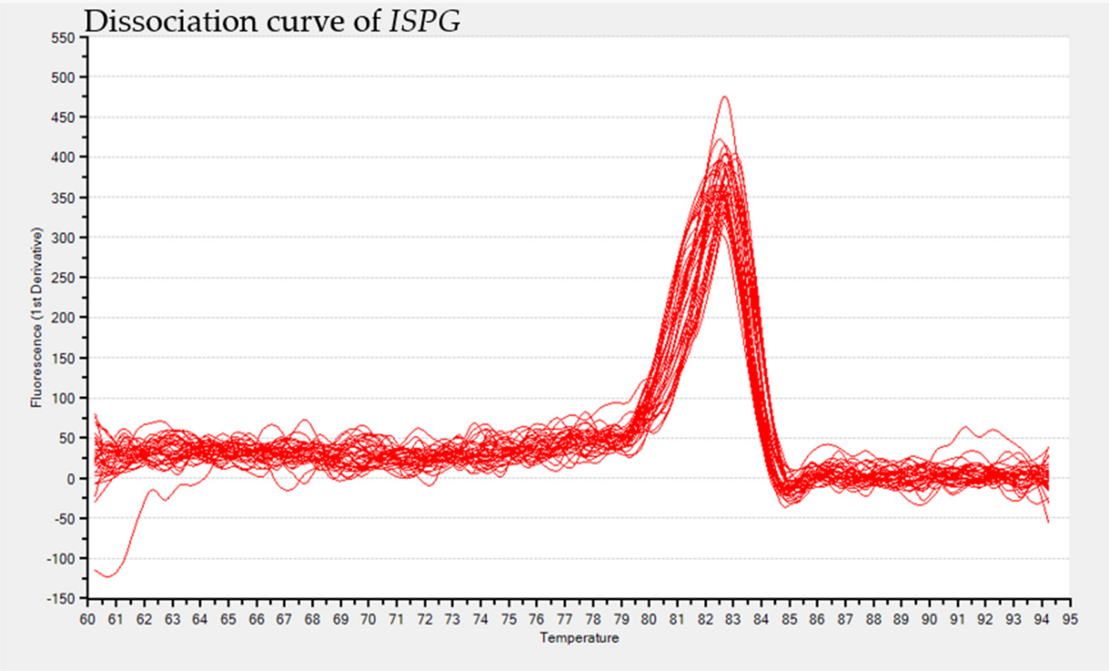

Figure S23. Dissociation curve of *ISPG*.

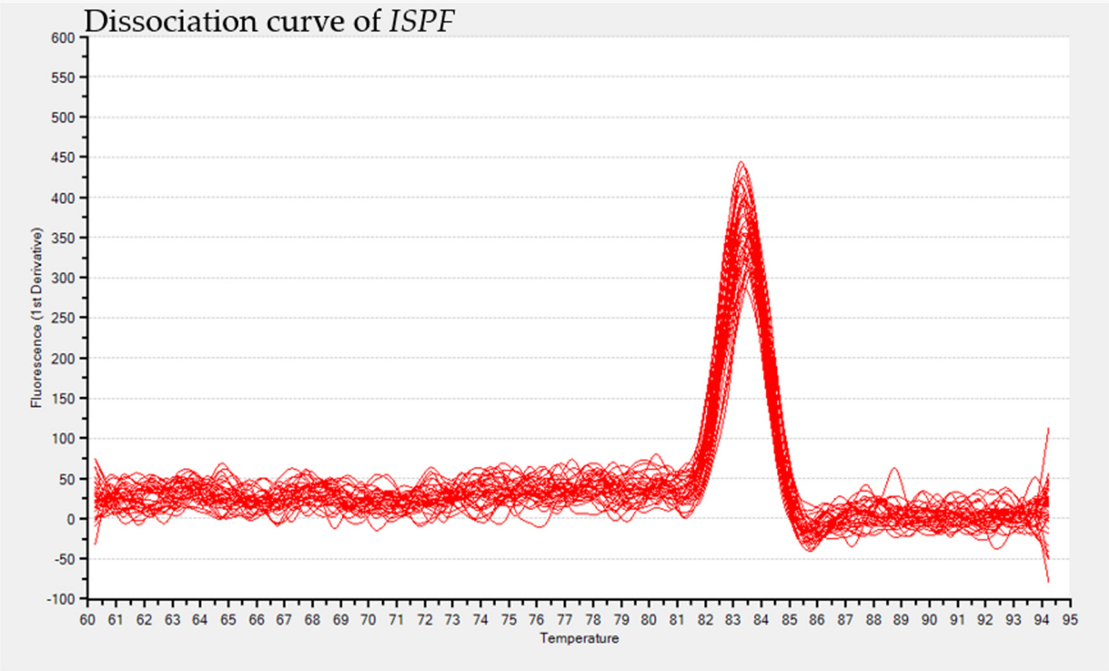

Figure S24. Dissociation curve of *ISPF*.

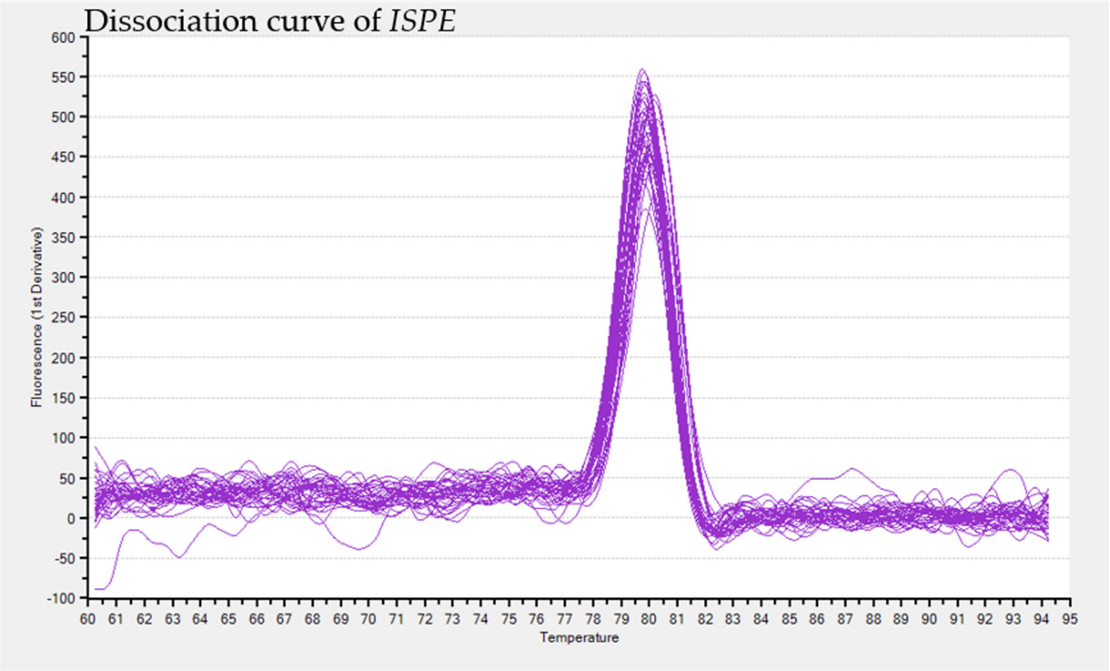

Figure S25. Dissociation curve of *ISPE*.

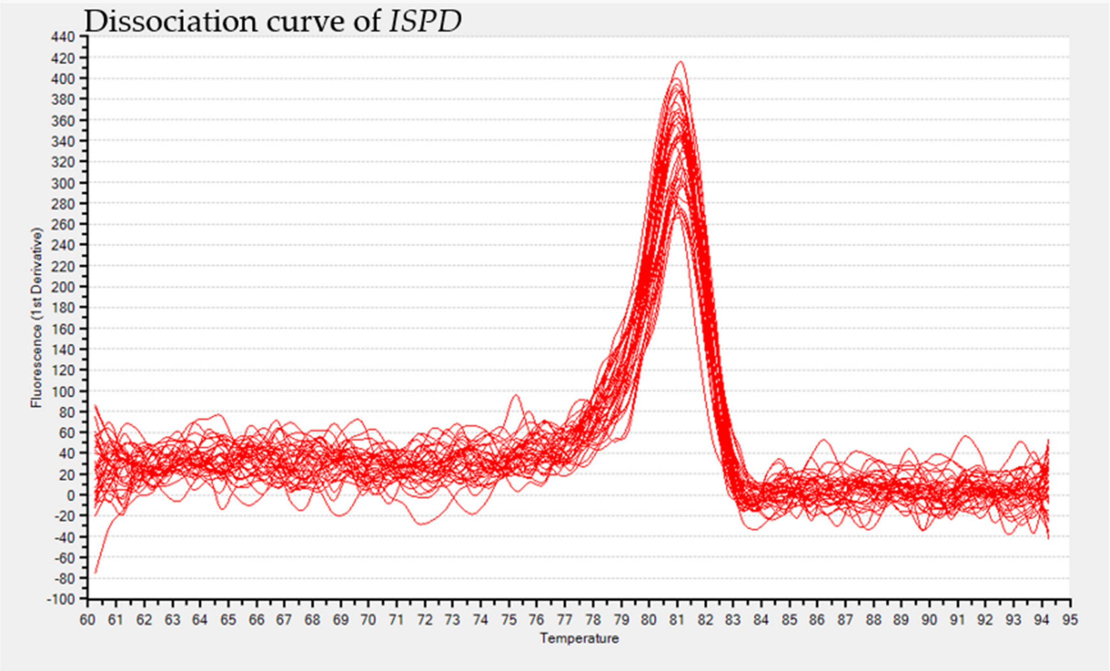

Figure S26. Dissociation curve of *ISPD*.

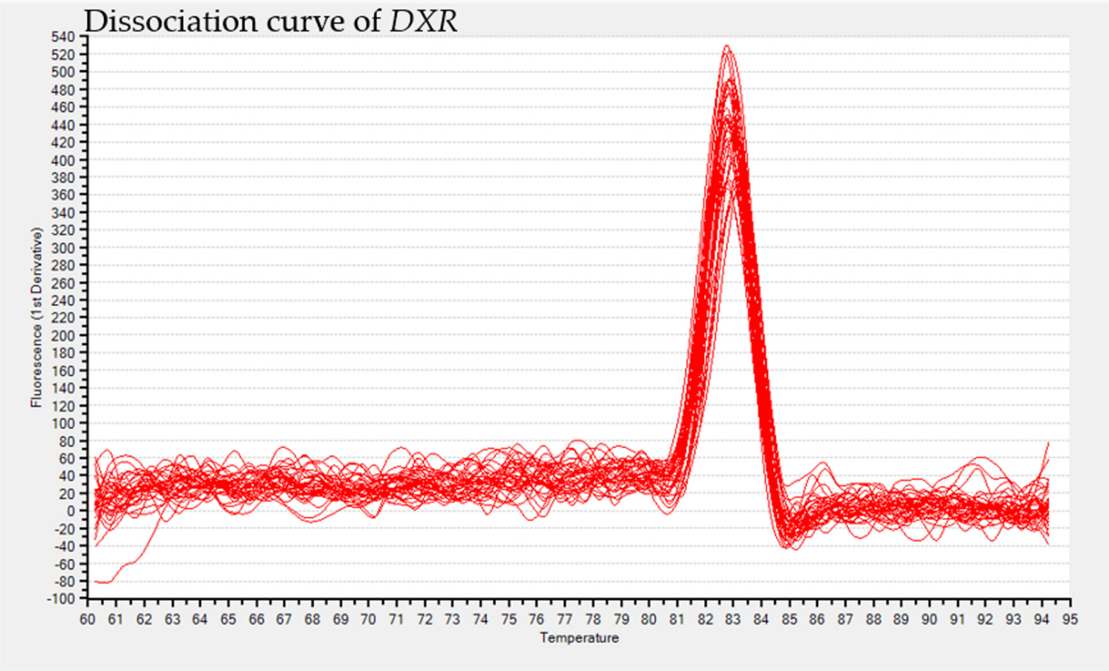

Figure S27. Dissociation curve of *DXR*.

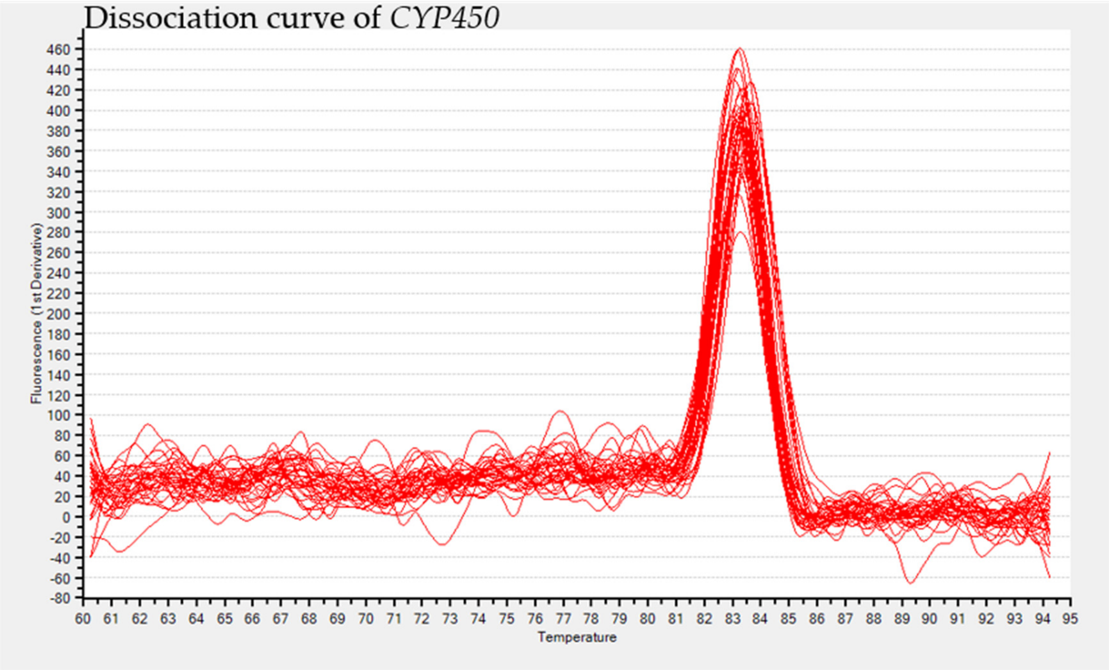

Figure S28. Dissociation curve of *CYP450*.
